# A cytoplasmic motif in HLA-E that drives clathrin-mediated endocytosis and VCP-associated postendocytic trafficking

**DOI:** 10.1073/pnas.2514956122

**Published:** 2025-10-24

**Authors:** Wanlin He, Andreas Damianou, Iolanda Vendrell, Klaus Früh, Daowen I. Yin, Frances M. Brodsky, Benedikt M. Kessler, Simon Brackenridge, Persephone Borrow, Geraldine M. Gillespie, Andrew J. McMichael

**Affiliations:** ^a^Center for Immuno-Oncology, Nuffield Department of Medicine, University of Oxford, Oxford OX3 7DQ, United Kingdom; ^b^State Key Laboratory of Oral Diseases and National Center for Stomatology and National Clinical Research Center for Oral Diseases, West China Hospital of Stomatology, Sichuan University, Chengdu 610041, China; ^c^Target Discovery Institute, Centre for Medicines Discovery, Nuffield Department of Medicine, University of Oxford, Oxford OX3 7FZ, United Kingdom; ^d^Chinese Academy of Medical Sciences Oxford Institute, Nuffield Department of Medicine, University of Oxford, Oxford OX3 7BN, United Kingdom; ^e^Vaccine and Gene Therapy Institute and Division of Pathology and Immunology, Oregon National Primate Research Center, Oregon Health and Science University, Beaverton, OR 97006; ^f^Department of Structural and Molecular Biology, Division of Biosciences, University College London, London WC1E 6BT, United Kingdom; ^g^Department of Structural and Molecular Biology, Institute of Structural and Molecular Biology, Birkbeck and University College London, London WC1E 7HX, United Kingdom

**Keywords:** HLA-E, nonclassical MHC class I, cytoplasmic tail, clathrin-mediated endocytosis, valosin-containing protein (VCP)

## Abstract

Human Leukocyte Antigen E (HLA-E) is a nonclassical MHC class I molecule that exhibits dual immunological functions in regulating natural killer (NK) cells and T cells through unusual trafficking patterns. We previously reported that HLA-E surface expression is low and transient due to its cytoplasmic tail and dominant VL9 peptide, making it a dynamic indicator of cellular status for NK cell surveillance. Here, we identify a sequence motif in the HLA-E cytoplasmic tail that enables rapid internalization via clathrin-mediated endocytosis (CME) through interaction with the adaptor protein 2 (AP-2) complex. Following internalization, HLA-E is routed to endosomes, where the same cytoplasmic motif and peptide loading together facilitate its reappearance on the cell surface—a process influenced by valosin-containing protein (VCP). Our findings reveal previously unrecognized endosomal trafficking pathways and regulatory mechanisms that distinguish HLA-E from classical HLA class I molecules, with broad implications for understanding the immunoregulatory roles of HLA-E.

## Significance

By presenting a conserved self-peptide to NKG2A/C-CD94 receptors on NK cells, nonpolymorphic HLA-E plays a central role in regulating innate immunity. While HLA-E can also present foreign peptides to stimulate protective T cell responses, these are naturally rare and subdominant to classical T cell responses. Here, we identify a lysine/tryptophan-based motif in the HLA-E cytoplasmic tail that facilitates rapid surface turnover via clathrin-mediated endocytosis. This motif, combined with peptide binding, supports rapid surface reappearance of HLA-E, a process modulated by the AAA ATPase VCP. These insights into the cell biology of HLA-E open broad avenues for enhancing these noncanonical T cell responses, offering promising opportunities for universal immunotherapies.

Major histocompatibility complex (MHC)-E is a nonclassical MHC class Ib molecule, whose primary role is to present the leader sequence peptides from MHC class Ia molecules, typically VMAPRT(L/V)(V/L/I/F)L (VL9) (1, 2). The MHC-E-VL9 complex is recognized by the NKG2x/CD94 receptors on natural killer (NK) cells, thereby monitoring the integrity of the MHC-I antigen presentation pathway and regulating NK cell activation (3, 4). Human Leukocyte Antigen E (HLA-E, human MHC-E) has only two common alleles (HLA-E*01:01 and HLA-E*01:03), which share an identical peptide-binding groove that is optimized for VL9 binding (5). The surface level of HLA-E is low and transient (6), providing a dynamic and temporal representation of MHC-I availability. The limited polymorphism and unconventional trafficking patterns of HLA-E align with its primary role in regulating innate immunity and reflect evolutionary selection pressures distinct from those shaping the highly polymorphic classical MHC I molecules.

Beyond its role in innate immune regulation, HLA-E can also present foreign or altered self-peptides to prime T cells (7–10). Such HLA-E-restricted T cell responses have been observed in several acute infections, including (HIV)-1 (11), human cytomegalovirus (HCMV) (12, 13), Epstein–Barr virus (EBV) (14), SARS-CoV-2 (15), *Mycobacterium tuberculosis* (Mtb) (16, 17), and *Salmonella enterica* serotype Typhi (S.Typhi) (18, 19). While the classical MHC-I pathways are often compromised by pathogens in these contexts (20), HLA-E is often resistant to downregulation and even upregulated, enabling effective HLA-E-restricted T cell responses (21, 22). Similar considerations have been implicated in several cancers (23). These studies suggest that the unusual trafficking of HLA-E plays a critical role in both NK and T cell regulation. Understanding these processes could provide options for immunotherapies targeting HLA-E in infections and cancers.

Here, we show that surface HLA-E undergoes rapid clathrin-mediated endocytosis (CME) via a cytoplasmic motif that recruits AP-2 through its μ2 subunit. Internalized HLA-E can rapidly reappear on the cell surface, a process that requires both the cytoplasmic motif and peptide loading. The valosin-containing protein (VCP) also impacts this process. These findings therefore demonstrate how HLA-E surface expression is dynamically regulated for precise NK cell regulation. The unconventional endosomal pathways identified here reveal how HLA-E acquires foreign peptides and primes unusual T cell responses.

## Results

### A Cytoplasmic Lysine/Tryptophan-Based Motif Promotes Rapid Internalization of HLA-E.

**Fig 1.**
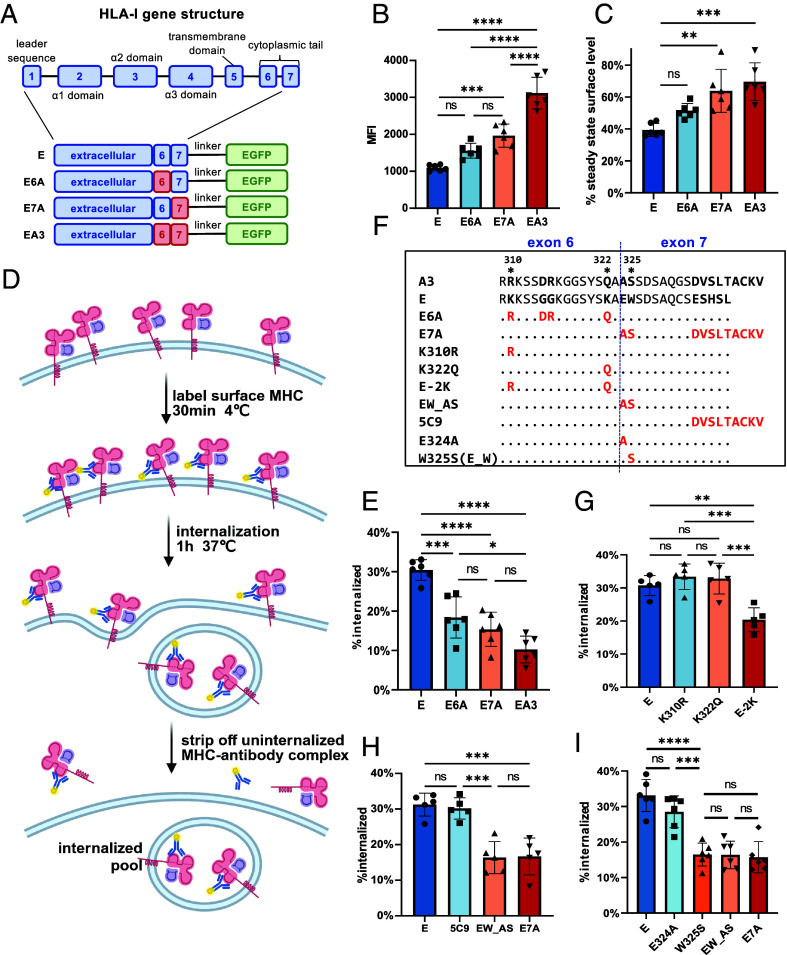
Rapid internalization of HLA-E relies on a lysine/tryptophan-based motif in the cytoplasmic tail. (*A*) Schematic representation of the HLA-E gene structure and HLA-E constructs with different cytoplasmic tails tagged with EGFP at the C-terminus. Exons from HLA-E and HLA-A3 are shown in blue and red, respectively. (*B*) Mean fluorescence intensity (MFI) of surface HLA-E staining in HEK293T cells transiently transfected with the HLA-E constructs shown in (*A*) (gated on EGFP+ cells). (*C*) BFA assay in HEK293T cells transiently transfected with different HLA-E constructs shown in (*A*). The average cell surface MFI before BFA addition was set to 100%, and the MFI after BFA incubation for 1 h was normalized as a percentage of this. (*D*) Diagram illustrating the internalization assay (created with BioRender.com). (*E*) Internalization assay in HEK293T cells transiently transfected with different HLA-E constructs shown in (*A*). The MFI of antibody-labeled cells without acid stripping was set to 100%, and the MFI of antibody-labeled cells with acid stripping (but without internalization) was set to 0%. The percentage of HLA-E internalized after 1 h was normalized accordingly. (*F*) Sequences of the cytoplasmic tail of different constructs. Sequence identity is indicated by dots and mutated positions are highlighted in red. (*G*–*I*) Internalization assay in HEK293T cells transiently transfected with different HLA-E constructs from (*F*), plotted as described for (*E*). Data were collected for five (*G* and *H*) or six (*B*, *C*, *E*, and *I*) replicates and are shown as mean ± SD (error bars). Statistical analysis was performed using one-way ANOVA with Tukey’s post hoc test. Asterisks indicate the statistical significance of differences between indicated groups: ns, not significant; *P < 0.05; **P < 0.01; ***P < 0.001; ****P < 0.0001.

We previously reported that the rapid internalization of HLA-E depends on its cytoplasmic tail (6). Since HLA-I’s cytoplasmic tails consist of exon 6 and exon 7 (*SI Appendix*, Fig. S1), we created HLA-E exon-swap mutants with HLA-A3, generating constructs E6A, E7A, and EA3 (Fig. 1*A*). While single exon replacements only slightly increased HLA-E surface expression, replacing both (EA3) showed significant elevation (Fig. 1*B* and *SI Appendix*, Fig. S2). Brefeldin A (BFA) decay assays showed that both exons contribute to HLA-E’s rapid surface decay (Fig. 1*C* and *SI Appendix*, Fig. S3*A*). Acid stripping assays further revealed that around 30% of HLA-E was internalized within 1 h, compared to less than 20% for the mutants (Fig. 1 *D* and *E* and *SI Appendix*, Fig. S3 *B* and *C*). These results indicate that the rapid internalization of HLA-E requires both exon 6 and 7 of its cytoplasmic tail.

Next, we examined exon 6 and 7 separately to identify potential motifs. Although exon 6 is conserved across HLA-I molecules, HLA-E possesses two unique lysines at positions 310 and 322 (Fig. 1*F* and *SI Appendix*, Fig. S1). While individual mutations (K310R, K322Q) had minimal effects, mutating both lysines (E-2 K) modestly increased HLA-E surface expression (*SI Appendix*, Fig. S4*A*). K310R and K322Q had similar surface lifetimes and internalization rates as HLA-E, whereas E-2 K exhibited higher surface stability and slower internalization (Fig. 1*G* and *SI Appendix*, Fig. S4 *B*–*D*). Therefore, either lysine alone is sufficient to drive HLA-E’s rapid internalization, indicating functional redundancy.

Exon 7 exhibits lower sequence conservation, with only the central region (positions 326 to 332) showing relatively high conservation (*SI Appendix*, Fig. S1). Replacing the first two (EW_AS) or last five (5C9) amino acids from exon 7 with HLA-A3 residues revealed that the first two amino acids are critical (Fig. 1 *F* and *H* and *SI Appendix*, Fig. S5). Single mutants (E324A, W325S) showed that W325S mimicked EW_AS and E7A in surface stability and internalization, while E324A resembled wild-type (Fig. 1 *F* and *I* and *SI Appendix*, Fig. S6). Thus, the tryptophan at position 325 drives exon 7’s internalization effect.

### The Lysine/Tryptophan-Based Motif Is Sufficient to Elicit Fast Internalization and Endosomal Enrichment of HLA-I Molecules.

**Fig 2.**
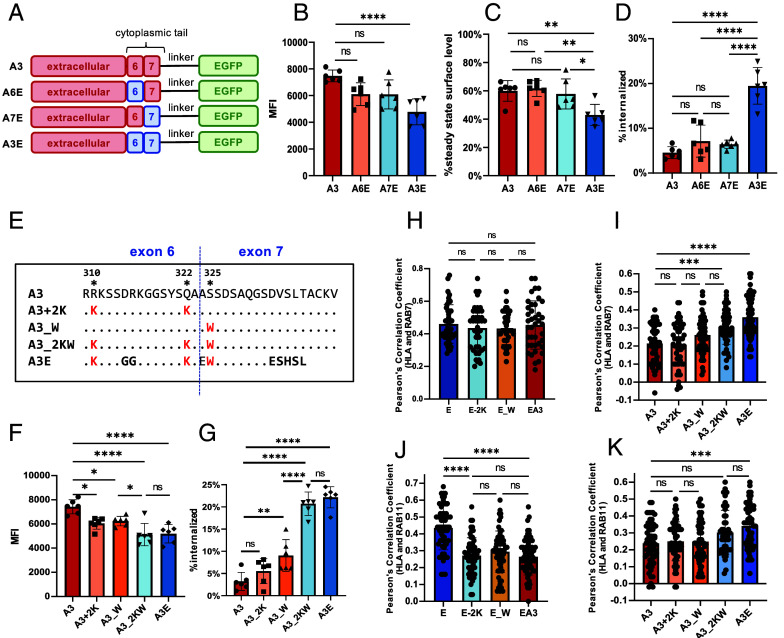
The lysine/tryptophan-based motif in the cytoplasmic tail is sufficient to drive the fast internalization and endosomal enrichment of HLA-A3. (*A*) Schematic representation of HLA-A3 constructs with different cytoplasmic tails tagged with EGFP at the C-terminus. Exons from HLA-E and HLA-A3 are shown in blue and red, respectively. (*B*) MFI of surface HLA-E staining in HEK293T cells transiently transfected with the HLA-A3 constructs shown in (*A*) (gated on EGFP+ cells). (*C*) BFA assay in HEK293T cells transiently transfected with different HLA-A3 constructs shown in (*A*). The average cell surface MFI before BFA addition was set to 100%, and the MFI after BFA incubation for 4 h was normalized as a percentage of this. (*D*) Internalization assay in HEK293T cells transiently transfected with different HLA-A3 constructs shown in (*A*). The MFI of antibody-labeled cells without acid stripping was set to 100%, and the MFI of antibody-labeled cells with acid stripping (but without internalization) was set to 0%. The percentage of HLA-A3 internalized after 1 h was normalized accordingly. (*E*) Sequences of the cytoplasmic tail of different constructs. Sequence identity is indicated by dots and mutated positions are highlighted in red. (*F*) MFI of surface HLA-E staining in HEK293T cells transiently transfected with the HLA-A3 constructs shown in (*E*) (gated on EGFP+ cells). (*G*) Internalization assay in HEK293T cells transiently transfected with different HLA-A3 constructs from (*E*), plotted as described in (*D*). Data in *B*–*D*, *F*, and *G* were collected for six replicates and are shown as mean ± SD (error bars). (*H*–*K*) Colocalization of different HLA-E (*H* and *J*) and HLA-A3 (*I* and *K*) constructs with late endosomes (RAB7, *H* and *I*) or recycling endosomes (Rab11, *J* and *K*) in HeLa cells. Colocalization was quantified by the calculation of the Pearson’s correlation coefficient value for each cell, with 40 to 60 cells per sample. Statistical analysis was performed using one-way ANOVA with Tukey’s post hoc test. Asterisks indicate the statistical significance of differences between indicated groups: ns, not significant; *P < 0.05; **P < 0.01; ***P < 0.001; ****P < 0.0001.

To investigate whether this lysine/tryptophan motif drives fast surface turnover in other HLA-I molecules, we replaced exon 6 or exon 7 of HLA-A3 with those of HLA-E, creating A6E and A7E constructs (Fig. 2*A*). While surface expression, stability, and internalization rates of A6E and A7E were similar to HLA-A3 (Fig. 2 *B*–*D* and *SI Appendix*, Fig. S7 *A*, *B*, and *D*), swapping both exons (A3E) increased the internalization rate from 10% to around 20%, indicating that both exons are required to promote internalization. While introducing either the two lysines (A3 + 2K) or the tryptophan (A3_W) into HLA-A3 reduced its surface levels and internalization rates, the presence of both mutations (A3_2KW) was necessary to match the reduced levels observed for HLA-A3E (Fig. 2 *E*–*G* and *SI Appendix*, Fig. S7 *C* and *E*). These findings indicate that the lysine/tryptophan-based motif is both necessary and sufficient to facilitate the rapid internalization of HLA-I molecules.

Given that the HLA-E cytoplasmic tail can facilitate the enrichment of HLA-A3 within late and recycling endosomes (6), we analyzed how the lysine/tryptophan-based motif influences the endosomal distribution of HLA-I molecules (Fig. 2 *H*-*K* and *SI Appendix*, Fig. S8). While no differences in late endosome localization were observed among HLA-E variants (Fig. 2*H* and *SI Appendix*, Fig. S8*C*), the entire motif significantly increased HLA-A3 levels in late endosomes (Fig. 2*I* and *SI Appendix*, Fig. S8*A*). HLA-E enrichment in recycling endosomes was diminished when either lysines or tryptophan were missing. (Fig. 2*J* and *SI Appendix*, Fig. S8*D*). No significant changes in recycling endosome localization were observed between HLA-A3, A3+2 K, A3_W, and A3_2KW (Fig. 2*K* and *SI Appendix*, Fig. S8*B*). These results highlight the critical role of the lysine/tryptophan-based motif in promoting HLA-I enrichment in late and recycling endosomes, though additional factors also contribute to this process.

### Efficient HLA-E Surface Reappearance Requires the Lysine/Tryptophan Motif and Strong Binding Peptides.

**Fig 3.**
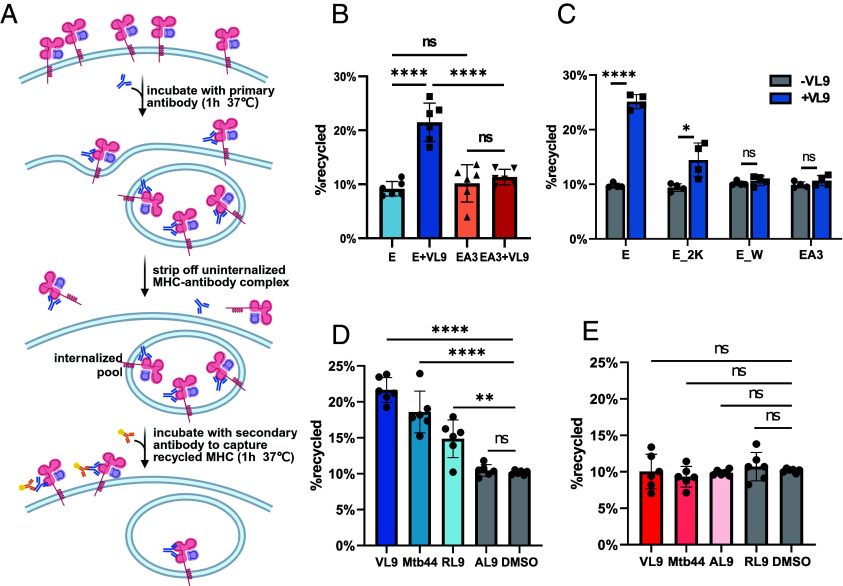
The lysine/tryptophan-based motif and strong binding peptides together promote HLA-E recycling. (*A*) Diagram illustrating the recycling assay (created with BioRender.com). (*B*) Recycling assay in HEK293T cells transiently transfected with HLA-E (E) or HLA-EA3 (EA3) with or without VL9 peptide pulsing (50 µM final). The total internalized mAb-bound HLA-E after acid stripping was assessed by intracellular staining and was set to 100%. The MFI of antibody-labeled cells with acid stripping (but without recycling) was set to 0%. The percentage of HLA-E recycled after 1 h was normalized accordingly. (*C*) Recycling assay in HEK293T cells transiently transfected with HLA-E WT (E), or versions with two lysines (E _ 2 K), tryptophan (E _ W), or both (E _ 2KW) mutated. HLA-EA3 was used as control. (*D* and *E*) Recycling assay in HEK293T cells transiently transfected with HLA-E (*D*) or HLA-EA3 (*E*) in the presence of peptides of different affinity. Data are plotted as in (*B*). Data were collected for four (*C*) or six (*B*, *D*, and *E*) replicates and are shown as mean ± SD (error bars). Statistical analysis was performed using one-way ANOVA with Tukey’s post hoc test (*B*, *D*, and *E*) or paired two-tailed *t* test with Welch’s correction (*C*). Asterisks indicate the statistical significance of differences between indicated groups: ns, not significant; *P < 0.05; **P < 0.001; ****P < 0.0001.

We next examined how the cytoplasmic tail and peptide binding influence the postendocytic fate of HLA-E (6). Using a previously established assay (Fig. 3*A*) (24), internalized HLA-E was labeled with an unconjugated primary antibody, and its reappearance at the cell surface was detected by a fluorophore-tagged secondary antibody added during the recovering phase. This approach enables detection of HLA-E that returns to the surface, regardless of subsequent reinternalization. Without peptide addition, HLA-E and HLA-EA3 exhibited similar surface reappearance patterns (Fig. 3*B* and *SI Appendix*, Fig. S9*A*). However, VL9 peptide pulsing doubled HLA-E reappearance rates without affecting its internalization, while having no effect on HLA-EA3 (Fig. 3*B* and *SI Appendix*, Fig. S9 *A* and *B*). These results indicate that although the HLA-E cytoplasmic tail alone is sufficient to promote its rapid internalization, rapid surface reappearance requires both the tail and peptide binding.

To identify motifs contributing to this effect, we tested HLA-E mutants at internalization-critical residues (Fig. 1). With VL9 pulsing, around 25% of wild-type HLA-E reappeared on the cell surface within one hour, compared to around 15% for E _ 2 K and 10% for E _ W (Fig. 3*C*), suggesting that the lysine/tryptophan motif also drives efficient surface reappearance in the presence of strong binding peptides. We then assessed the impact of peptide affinity on this process (Fig. 3 *D* and *E*). The high-affinity Mtb44 and low-affinity RL9 peptides induced around 18% and 15% HLA-E surface reappearance, respectively (25), while the nonbinding AL9 peptide had no effect (6, 26) (Fig. 3*D*). None of these peptides affected HLA-EA3 (Fig. 3*E*). The role of peptide binding was confirmed using single-chain peptide-β2-microglobulin (β2 m)-MHC-I trimers (SCT) and dimers (SCD) for HLA-E and HLA-EA3 (*SI Appendix*, Fig. S9*C*) (26, 27).

In conclusion, efficient reappearance of HLA-E on the cell surface after internalization requires both its cytoplasmic tail and peptide binding. The lysine/tryptophan motif, while essential for HLA-E internalization, also contributes to its surface return. Peptides with higher affinity enhance this process more efficiently, likely by stabilizing the HLA-E complex. If the VL9 peptide dissociates during this process, alternative binding peptides, generated in lysosomes, could access HLA-E.

### HLA-E Proximity Proteome Includes COPII, CME, VCP, and ESCRT Complexes.

**Fig 4.**
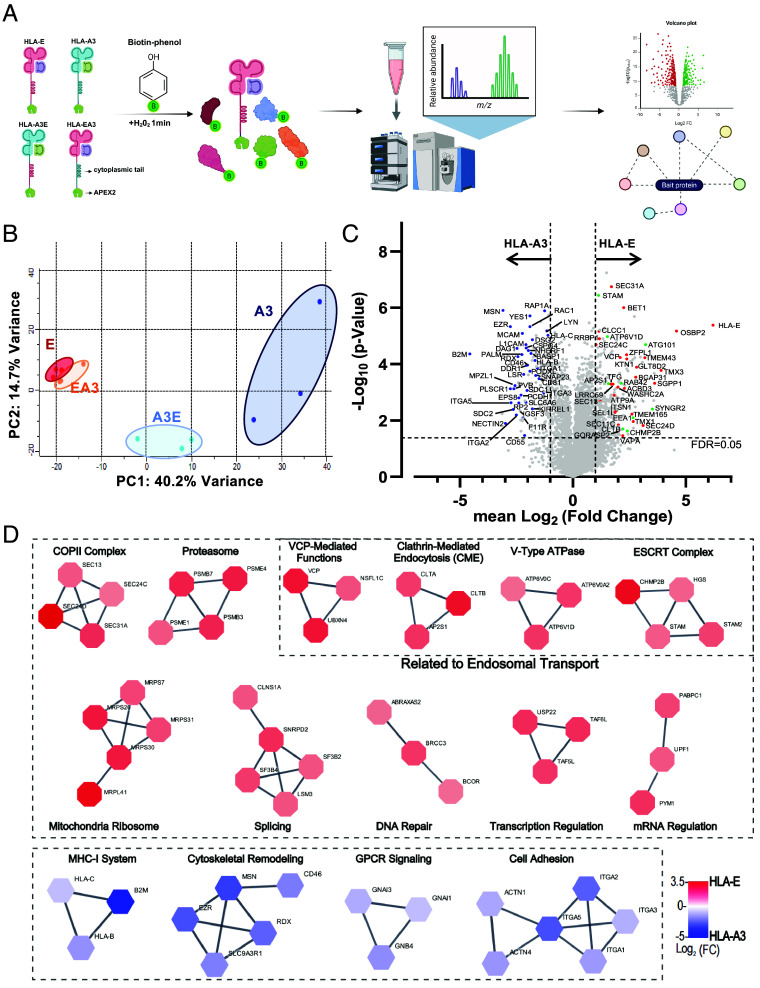
Proximity proteomics reveals the microenvironment of different HLA constructs. (*A*) Workflow of APEX2 proximity labeling followed by mass spectrometry analysis for HLA constructs (created with BioRender.com). (*B*) Principal Component (PC) analysis of 4 different HLA constructs, with 3 replicates for each. (*C*) Volcano plot showing fold changes (FCs) and false discovery rate (FDR)-adjusted *P*-values for relative protein quantification between HLA-E and HLA-A3. The most significant hits that primarily localized to ER-Golgi (red), endosomes (green), and plasma membrane (blue) are highlighted. Data are shown as means of three independent samples. (*D*) Protein–protein interaction networks from the STRING database (confidence cutoff 0.9) for proteins most significantly enriched in HLA-E-APEX2 (red) or HLA-A3-APEX2 (blue) experiments (FDR < 0.05 and FC ≥ 1). Cluster labels were added manually.

Due to HLA-E’s rapid turnover in the endosomal pathway, its interactions with regulators are likely transient. To identify pathways and proteins regulating the endosomal trafficking of HLA-E, we employed ascorbic acid peroxidase 2 (APEX2)-based proximity labeling to characterize its microenvironment, with HLA-A3 as the control and chimeric HLA-EA3 and HLA-A3E constructs to investigate the role of the cytoplasmic tail (Fig. 4*A*). Since C-terminal tagging does not affect HLA-E trafficking (6), APEX2 was fused to the C terminus of HLA constructs, followed by a FLAG tag (*SI Appendix*, Fig. S10*A*). Streptavidin-based immunoprecipitation (IP) and silver staining demonstrated robust biotin labeling and successful enrichment of biotinylated proteins (*SI Appendix*, Fig. S10 *B* and *C*). Immunofluorescence confirmed that APEX2 fusion did not alter the localization of these constructs (6), with biotinylated proteins matching their distribution patterns (*SI Appendix*, Fig. S10D). These results validated that APEX2 labeling accurately captures proximal proteins of different HLA microenvironments.

We then performed liquid chromatography–tandem mass spectrometry (LC–MS/MS) and identified 5,623 proteins, with 5,024 retained after filtering. Principal component analysis (PCA) revealed distinct clustering patterns for HLA constructs (Fig. 4*B*). HLA-EA3 patterns aligned closely with HLA-E, reflecting their predominant ER localization and low surface expression, while HLA-A3E exhibited an intermediate pattern between HLA-A3 and HLA-E. Comparing HLA-E and HLA-A3 proximity proteomes (Fig. 4*C*), HLA-A3-enriched proteins localized predominantly to the plasma membrane, while HLA-E-enriched proteins were mainly confined to ER-Golgi pathways and endosomes. Notably, β2 m was significantly more associated with HLA-A3 than HLA-E, likely due to HLA-E’s predominant ER localization, where proper association with β2 m is limited (28). Additionally, HLA-B and HLA-C were more enriched in HLA-A3, reflecting their overlapping spatial distribution. These findings highlight the distinct spatial and functional niches of HLA-E and HLA-A3, underscoring the specificity and biological relevance of their proximity proteomes.

Protein–protein interaction (PPI) network analysis of top hits revealed HLA-A3-enriched complexes linked to plasma membrane functions, including the MHC-I system, cytoskeletal remodeling, G protein–coupled receptor (GPCR) signaling, and cell adhesion (Fig. 4*D*). HLA-E-enriched complexes were associated with protein transport pathways, including coat protein complex II (COPII)-mediated ER-Golgi transport, CME, V-type ATPase for endosome acidification, and the endosomal sorting complex required for transport (ESCRT). The VCP-NSFL1C-UBXN4 complex, consisting of VCP, NSF-like protein 1C (NSFL1C), and UBX domain-containing protein 4 (UBXN4), which is critical for endoplasmic reticulum-associated degradation (ERAD), membrane remodeling, and autophagy (29), was also enriched. Protein complexes associated with DNA, transcription, splicing, and mitochondria represent common nonspecific background signals in APEX2 labeling (30, 31).

### siRNA Screening Combined with APEX2 Proximity Labeling Identifies Protein Networks Regulating the Endocytic Transport of HLA-E.

**Fig 5.**
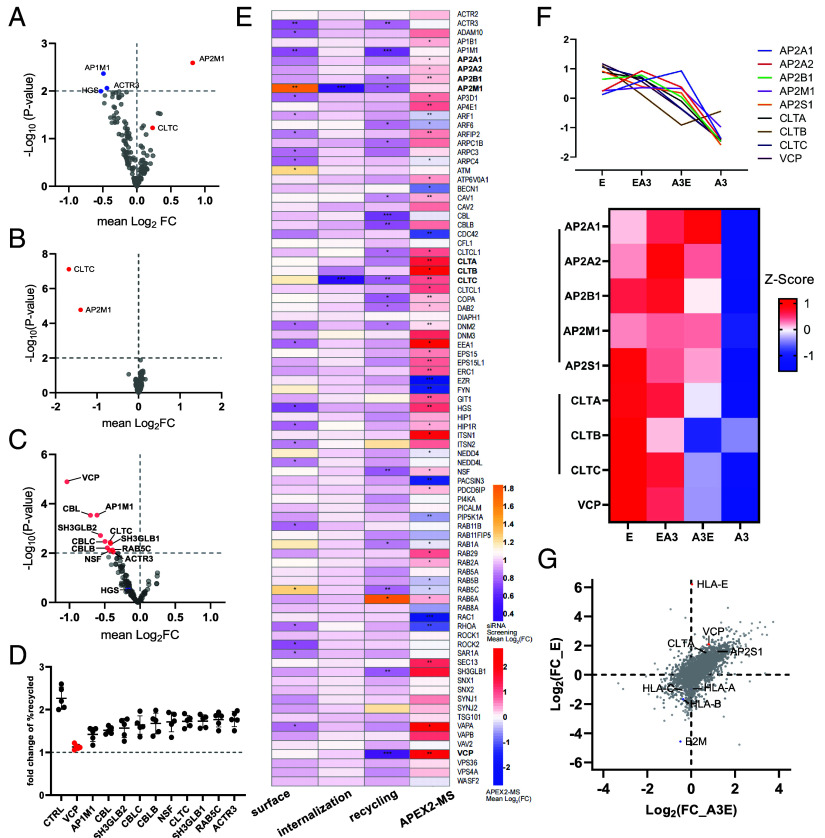
siRNA screening and proximity labeling identifies protein networks regulating HLA-E endosomal transport. (*A*) Volcano plot depicting the FC in relative surface HLA-E expression in THP-1 cells with siRNA-mediated depletion of various proteins relative to expression in control siRNA-treated cells, and the unadjusted *P*-value for each siRNA treatment. Each dot represents the average of three replicates for each target. Statistical analysis was performed using a one-sample Student’s *t* test, and the targets with a *P*-value < 0.01 were considered as hits and were highlighted in blue (downregulation) or red (upregulation). The CLTC target is also highlighted. (*B*) Volcano plot depicting the FC in relative internalization rate of HLA-E after 1 h in HEK293T cells with siRNA depletion of different proteins relative to that in control siRNA-treated cells, and the unadjusted *P*-value for each siRNA treatment. Internalization assays were carried out as in Fig. 1*D*. Each dot represents the average of five replicates for each target. Statistical analysis was performed using one-sample Student’s *t* test, and the targets with a *P*-value < 0.01 were considered as a hit and were highlighted in red. (*C*) Volcano plot depicting the FC for relative recycling rate of HLA-E after 1 h in HEK293T cells with siRNA depletion of different proteins relative to that in control siRNA-treated cells, and the unadjusted *P*-value for each siRNA treatment. Recycling assays were carried out as Fig. 3*A*. The recycling-promoting effect was assessed by calculating the FC increase of the percentage of HLA-E recycled with VL9 pulsing relative to the percentage without VL9. Each dot represents the average of five replicates for each target. Statistical analysis was performed using one-sample Student’s *t* test, and the targets with a *P*-value < 0.01 were considered as a hit and are highlighted in red. (*D*) FC increase in HLA-E recycled with VL9 pulsing for the significant hits from (C). Data are shown as mean ± SD (error bars), and the top hit VCP is highlighted in red. (*E*) Heatmap combining FCs and unadjusted (siRNA screens, Fig. 5 *A*-*C*) or FDR-adjusted (proximity labeling, Fig. 4*D*) *P* values from different assays. Statistical significance is represented as follows: *P < 0.05; **P < 0.01; ***P < 0.001; ****P < 0.0001. VCP and CME-associated proteins are highlighted in bold. (*F*) Heatmap showing the Z-scored expression levels of VCP and CME-associated proteins from the proximity labeling experiment, standardized to their mean across samples for the four HLA APEX2 samples. (*G*) Volcano plot showing FC in relative protein quantification for HLA-E and HLA-A3E against HLA-A3. Some significant hits enriched (red) or depleted (blue) for HLA-E are highlighted. Data are shown as means of three independent samples.

APEX2 proximity labeling identifies proteins based on proximity rather than direct interactions, limiting functional insights. To identify proteins and pathways involved in HLA-E plasma membrane trafficking, we performed a siRNA screen targeting 140 membrane trafficking-associated proteins in THP-1 cells, using transient knockdown to minimize toxicity. Only *AP2M1* knockdown significantly increased HLA-E surface levels (Fig. 5*A*), suggesting a crucial role for AP-2 in the initial steps of HLA-E internalization. Several knockdowns led to reduced HLA-E surface levels, reflecting its multistep transport after internalization.

To examine if depletion of any proteins inhibits the internalization or surface return of HLA-E, we used HEK293T cells transfected with HLA-E for stronger signal detection due to its low endogenous expression in THP-1 cells. Knockdown of *CLTC* or *AP2M1* significantly inhibited HLA-E internalization (Fig. 5*B*), whereas depletion of other proteins, including ARF6, which is crucial for classical MHC-I endocytosis (32), had no effect. This implies that HLA-E is internalized via CME, a well-studied internalization pathway where AP-2 recognizes cargo sorting signals, recruits clathrin to form coated pits, and directs cargo to endosomal sorting pathways (33, 34). Inhibition of several proteins impaired HLA-E surface reappearance (Fig. 5*C*). Notably, VCP (p97) knockdown led to a near-complete loss of surface recovery, while other knockdowns only caused partial inhibition (Fig. 5*D*). VCP is an AAA ATPase essential for diverse cellular processes, including ERAD, ubiquitin-dependent protein degradation, phagosome-derived protein translocation, autophagosome maturation, and endolysosomal sorting (35–37).

We then integrated siRNA screens with proximity labeling data to identify hits with potential biological impact on HLA-E trafficking. Of the 140 proteins from the siRNA library, 84 appeared in the HLA-E proximity proteome (Fig. 5*E*). Notably, proteins associated with the AP-2 complex, clathrin-coated vesicles (CCVs), and VCP were enriched in the HLA-E proximitome compared to HLA-A3. This aligns with PPI network analysis, which highlighted CME and VCP-related pathways in the HLA-E microenvironment (Fig. 4*D*). To compare the proximity labeling of these potential targets across HLA variants, we normalized the data using Z-scores (Fig. 5*F*). Proteins associated with AP-2, CCVs, and VCP were highly enriched in HLA-E but low in HLA-A3, with HLA-A3E showing intermediate levels (Fig. 5*F*), implicating HLA-E cytoplasmic tail’s contribution to their enrichment. Comparative analysis, using HLA-A3 as a reference, revealed strong correlations between HLA-E and HLA-A3E proximitomes, with CLTA, AP2S1, and VCP enriched in both (Fig. 5*G*).

In summary, siRNA screens and proximity labeling revealed that the HLA-E cytoplasmic tail likely drives rapid internalization via CME, mediated by AP-2 and clathrin, and promotes fast surface return via a pathway that is modulated by VCP.

### Surface HLA-E Is Rapidly Internalized Via CME.

**Fig 6.**
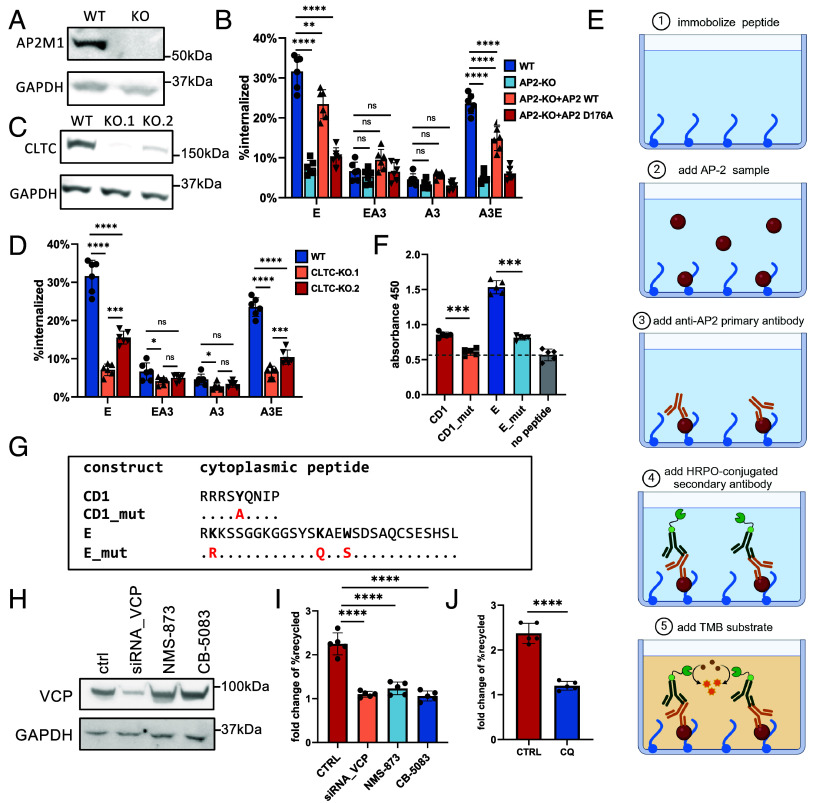
The unconventional endosomal transport of HLA-E depends on CME and VCP-dependent surface return. (*A*) Immunoblot for AP2M1 in HEK293T cells with *AP2M1* knocked out. (*B*) HLA internalization rates after 1 h in WT HEK293T cells or HEK293T cells with *AP2M1* knockout (*AP2*-KO), which were transiently transfected with the indicated HLA constructs, or cotransfected with HLA constructs and WT AP2M1 (AP2 WT) or its dominant-negative mutant (AP2 D176A). Internalization assays were carried out as described in Fig. 1*D*. (*C*) Immunoblot for CLTC in HEK293T cells with *CLTC* knocked out with two different gRNAs (KO.1, KO.2). (*D*) HLA internalization rates after 1 h in WT HEK293T cells or HEK293T cells with *CLTC* knocked out were transiently transfected with the indicated HLA constructs. Internalization assays were carried out as described in Fig. 1*D*. (*E*) Diagram illustrating the ELISA-based peptide binding assay (created with BioRender.com). (*F*) The binding of the cytoplasmic tails of CD1, HLA-E, and their respective mutated versions lacking the binding motif to the AP-2 complex was assessed by ELISA, as illustrated in *E*. Data were collected for five replicates (three technical repeats/run) and are shown as mean ± SEM. (*G*) Sequences of the cytoplasmic tail of different constructs. Sequence identity is indicated by dots and mutated positions are highlighted in red. (*H*) Immunoblot for VCP in HEK293T cells with siRNA-mediated *VCP* knockdown or after overnight incubation with NMS-873 (5 nM) and CB-5083 (2 nM). (*I*) Recycling of HLA-E after 1 h in HEK293T cells with siRNA-mediated VCP depletion or following overnight incubation with NMS-873 (5 nM) or CB-5083 (2 nM) with or without VL9 peptide (50 µM) during internalization. Recycling assays were carried out as in Fig. 3*A*. (*J*) Recycling of HLA-E after 1 h in HEK293T cells incubated with chloroquine (CQ, 20 µM) overnight with or without VL9 peptide (50 µM) during internalization. Recycling assays were carried out as Fig. 3*A*. Data were collected from six (*B* and *D*) or five (*H* and *I*) replicates and are presented as mean ± SD (error bars). Statistical analyses were performed using one-way ANOVA with Tukey’s post hoc test (*B*, *D*, and *H*) or paired two-tailed *t* test with Welch’s correction (F, I). Asterisks denote statistical significance: ns, not significant; *P < 0.05; **P < 0.01; ***P < 0.001; ****P < 0.0001.

The μ2 subunit of AP-2, encoded by *AP2M*1, interacts with cytoplasmic internalization signals in cargo proteins, with the YXXØ motif (Ø = bulky hydrophobic residue, X = any residue) being the most studied (38, 39). Mutation of the aspartic acid at position 176 (D176A) in the μ2 binding pocket disrupts motif interaction and impairs endocytosis (40, 41). To test whether the lysine/tryptophan-based motif is similarly recognized by AP-2, we generated *AP2M1* knock-out (KO) HEK293T cells (Fig. 6*A*). HLA-E and HLA-A3E showed rapid internalization in WT cells but not in *AP2M1*-KO cells (*AP2*-KO+NONE) (Fig. 6*B* and *SI Appendix*, Fig. S11*A*). Transfecting *AP2M1*-KO cells with WT *AP2M1* (*AP2*-KO+AP2 WT) partially restored internalization, whereas the D176 mutant (*AP2*-KO+AP2 D176A) had no effect, suggesting that the lysine/tryptophan-based motif likely interacts with the μ2 subunit, similar to the classical YXXØ motif. To further examine the role of clathrin, we generated two CLTC-KO cell lines (Fig. 6*C*), and both showed impaired endocytosis of HLA-E and HLA-A3E (Fig. 6*D* and *SI Appendix*, Fig. S11*B*). Unlike AP2M1, CLTC depletion did not significantly increase surface HLA-E levels (Fig. 5*A*), likely because of clathrin’s downstream role in CME or its involvement in multiple endocytic processes.

We then validated the interaction between AP-2 and the lysine/tryptophan-based motif using an enzyme-linked immunosorbent assay (ELISA)-based peptide binding assay (Fig. 6*E*). The HLA-E cytoplasmic tail peptide (E) bound significantly more strongly to AP-2 than its mutant counterpart lacking the lysine/tryptophan motif (E _ mut) (Fig. 6 *F* and *G*). Notably, the CD1b cytoplasmic tail peptide (CD1), which is known to interact with AP-2 via the YXXØ motif (42), showed only slightly stronger binding than its mutant (CD1_mut), suggesting that the newly identified lysine/tryptophan-based motif may engage AP-2 more strongly than the classical tyrosine-based motif.

In summary, unlike classical MHC-I molecules, HLA-E undergoes rapid internalization through CME, mediated by a distinctive lysine/tryptophan-based motif in its cytoplasmic tail that interacts with the μ2 subunit of AP-2, akin to the YXXØ motif.

### Rapid Surface Reappearance of HLA-E Requires VCP.

To validate VCP’s role in facilitating HLA-E surface return, we knocked down VCP in HEK293T cells using an independent siRNA and observed complete inhibition of HLA-E recycling (Fig. 6 *H* and *I*), excluding the possibility of off-target effects. Inhibiting VCP’s ATPase activity with NMS-873 or CB-5083, which specifically block VCP’s ATPase function via different mechanisms without altering its expression (43, 44) (Fig. 6*H*), also blocked its surface reappearance (Fig. 6*I* and *SI Appendix*, Fig. S12), confirming the necessity of its enzymatic function in this process. Given the role of VCP in endosomal protein sorting (45), we tested whether interference with the functions of endosomes with chloroquine (CQ) would affect this process. CQ treatment significantly inhibited the surface return of HLA-E (Fig. 6*J*), supporting the hypothesis that VCP’s ATPase activity drives endosomal sorting of HLA-E following internalization, which is required for its subsequent surface return. Therefore, while VCP’s primary role lies in ERAD and ubiquitin-dependent degradation, it also participates endosomal sorting; perhaps it is this latter function that is associated with its return to the cell surface.

## Discussion

In this study, we established the membrane trafficking pathways for HLA-E and the functional mediators that enable HLA-E to play a dual regulatory role in innate and adaptive immunity. We identified a lysine/tryptophan-based motif in the HLA-E cytoplasmic tail that facilitates CME through interaction with the μ2 subunit of the AP-2 complex. This motif, in conjunction with strong binding peptides, also promotes rapid HLA-E surface return via a VCP-dependent pathway. These findings demonstrate how HLA-E surface expression is tightly regulated for interactions with the CD94/NKG2x receptors on NK cells and some T cells. The routing of HLA-E to endosomal compartments for antigen processing and its subsequent recycling back to the cell surface, similar to HLA class II molecules, enables HLA-E to bind foreign peptides and prime unconventional T cell responses.

NK cell responsiveness is highly sensitive to HLA-E surface levels (3, 4). Although HLA-E displays low surface expression, it maintains a large ER reservoir, with surface translocation highly dependent on the availability of VL9 (6). Small fluctuations in classical HLA-I expression affect VL9 supply and consequently NK cell activation (46). Additionally, surface stability also regulates HLA-E’s surface levels. Our previous work showed that surface HLA-E is short-lived, which is controlled by its cytoplasmic tail (6). Here, we identified a lysine/tryptophan motif in the HLA-E cytoplasmic tail that interacts with the μ2 subunit of the AP-2 complex, thus driving CME. This trafficking mechanism resembles that of invariant chain-bound HLA-II, MR1, and CD1 molecules, but contrasts with the clathrin-independent, ARF6-dependent endocytic pathway employed by classical HLA-I molecules (47–52). CME is an ancient, well-conserved mechanism that enables rapid internalization of HLA-E, supporting its role in NK cell regulation (53, 54). In contrast, classical HLA-I and peptide-loaded HLA-II use clathrin-independent pathways, likely evolved to maintain their stable surface expression for T cell surveillance (55, 56).

Following internalization, HLA-E is directed to endosomal compartments, where it either undergoes degradation or, if structurally intact, acquires new peptides and recycles to the cell surface. Our in vitro studies have shown that VL9 has relatively weak binding affinity (25), likely an evolutionary adaptation for dynamic NK cell regulation. However, this also permits endosomal displacement of pathogen-derived peptides of lower affinity, which can sometimes prove beneficial for intracellular bacterial infections (16–18, 57–59). These features of HLA-E offer promising opportunities for vaccine and immunotherapy development, an area of increasing interest (60).

A rhesus cytomegalovirus (RhCMV)-vectored simian immunodeficiency virus (SIV) vaccine induces atypical Mamu-E (rhesus MHC-E)-restricted CD8+ T cells in vaccinated rhesus macaques (RMs) that can eradicate early SIV infection in 60% of challenged RMs (8, 9, 61, 62). This vaccine also induces MHC class II-restricted CD8+ T cell responses while lacking classical MHC-Ia-restricted responses (8). Although these MHC-II-restricted responses are nonprotective, their coelicitation implies shared antigen presentation pathways with MHC-E. This unusual MHC-E-restricted T cell priming requires viral-derived VL9 peptide provided by the Rh67 protein (63), which promotes MHC-E surface expression despite TAP blockade (64), enabling subsequent internalization and peptide exchange for SIV epitopes (9). Effective priming of CD8+ T cell responses appears to depend on relatively high amounts of peptide-loaded MHC-E in myeloid cells (57, 65), which is likely facilitated by blocking classical antigen presentation and supplying exogenous VL9.

We identified VCP as essential for the rapid reappearance of HLA-E at the cell surface, but the underlying mechanisms remain unclear. Ubiquitination likely plays a key role, as VCP recognizes ubiquitinated cargo for degradation or endosomal sorting (66). The presence of two unique lysines in HLA-E’s cytoplasmic motif suggests they may serve as potential recognition sites for VCP. CQ-mediated inhibition of HLA-E recycling further supports the involvement of endosomal sorting. Autophagosomes may also contribute, given the potential overlap between HLA-E endosomal trafficking and known MHC-II transport pathways (67), along with VCP’s established roles in autophagosome maturation and ubiquitinated protein degradation (35–37). Alternatively, VCP has been reported to be a clathrin-binding protein, so may influence a second clathrin-mediated trafficking pathway facilitating HLA-E transport (68). Moreover, internalized MHC molecules can undergo retrograde transport to the Golgi or even the ER (69). As VCP is required for protein translocation from phagosomal compartments for degradation, a process critical for cross-presentation (35), HLA-E may also acquire peptides beyond endosomes after endocytosis. Furthermore, considering VCP’s classical role in ERAD and the fact that many ER-localized HLA-E molecules are not properly folded, it is also plausible that VCP extracts misfolded HLA-E for degradation. Therefore, multiple pathways may coordinate the surface return of internalized HLA-E, with VCP likely acting at several stages. Further investigation into these mechanisms is required to fully understand how HLA-E acquires peptides and returns to the cell surface, which will be critical for the rational design of HLA-E-based immunotherapies.

In conclusion, our study characterizes the unconventional transport pathways and mechanisms through which HLA-E is internalized and subsequently returns to the cell surface after peptide loading. These findings reveal how the dynamic regulation of HLA-E surface expression could help balance NK cell activation and how its endosomal transport and peptide loading processes could enable HLA-E to bind foreign peptides in endosomes and induce unconventional yet potentially protective effector T cell responses. These unique properties position HLA-E as a pivotal player in both innate and adaptive immune regulation, offering significant potential for the development of immunotherapies and vaccines.

## Materials and Methods

### Peptides.

Synthetic peptides for peptide pulsing were reconstituted in dimethyl sulfoxide (DMSO) to a final concentration of 200 mM. N-terminally biotinylated peptides for ELISA-based peptide binding assay, incorporating an N-terminal protein linker, were dissolved in DPBS to a final concentration of 1 mg/mL. All peptides (85% purity, GenScript) were aliquoted and stored at −80 °C. Peptide sequences are listed in *SI Appendix*, Table S1.

### DNA constructs.

Plasmids expressing the WT AP2M1 (μ2-HA-WT,Addgene plasmid #32752; http://n2t.net/addgene:32752; RRID: Addgene_32752) or the mutated AP2M1 (μ2-HA-D176A, Addgene plasmid #32754; http://n2t.net/addgene:32754; RRID: Addgene_32754) were gifts from Alexander Sorkin.

Plasmids expressing HLA-E*01:03, HLA-A*03:01, or their cytoplasmic tail hybrids with the cytoplasmic tail (HLA-EA3 and HLA-A3E) were as previously described (6, 70). HLA-E*01:03-β2 m-peptide single-chain trimer construct (SCT) encoding VL9, RL9, and AL9, as well as the HLA-E*01:03-β2 m single-chain dimer construct (SCD), was generated as previously described (27). The corresponding SCT or SCD versions with the cytoplasmic tail swapped to HLA-A3 (HLA-EA3) were generated in the same way. Other constructs with manipulation in the cytoplasmic domain were created by inserting two fragments generated by overlap extension PCR using appropriate flanking primers (*SI Appendix*, Table S2) between the HindIII and NotI sites of pGEN using NEBuilder HiFi DNA Assembly (New England Biolabs) following the manufacturer’s instructions. Part 1 was amplified from the Part 1 backbone using pGEN F and Part 1, while Part 2 was amplified from the Part 2 backbone using pGEN TER R and Part 2 primer (*SI Appendix*, Table S3).

Lentivector plasmids expressing HLA-E, HLA-EA3, HLA-A3, or HLA-A3E with APEX2 and FLAG tag inserted consecutively at the C-terminus were generated by overlap extension PCR using appropriate flanking primers (*SI Appendix*, Table S2) and inserted between the BamHI and SalI sites of pLenti CMV GFP Puro [a gift from Eric Campeau and Paul Kaufman; Addgene plasmid # 17448 (71)] using NEBuilder HiFi DNA Assembly (New England Biolabs) following the manufacturer’s instructions. Part 1 and Part 2 were amplified from the corresponding primers and backbones listed in *SI Appendix*, Table S3. The APEX2-NLBP3-flag construct was generated in a previous study (31).

gRNA pairs targeting CLTC or AP2M1 were cloned into the lentiCRISPR v2 vector [a gift from Feng Zhang (Addgene plasmid # 52961; http://n2t.net/addgene:52961; RRID:Addgene_52961)] as previously described (72). sgRNA sequences are as follows:

AP2M1-sgRNA: 5’-GATGTCATCTCGGTAGACTC-3’;

CLTC-sgRNA-1: 5’-TGGCTTCAGTACCCTGACTA-3’;

CLTC-sgRNA-2: 5’-AAAAGTAGGAGAGCAGGCCC-3’.

### Cell Culture.

HEK293T and HeLa cells were cultured in DMEM (Life Technologies), while THP-1 wells were cultured in RPMI 1640 medium (Life Technologies). All culture media were supplemented with 10% heat-inactivated fetal bovine serum (Sigma) and penicillin/streptomycin (50 units/mL and 50 µg/mL, respectively; Life Technologies), and cells were maintained at 5% CO_2_/37 °C. 293 T KO cell lines and HeLa cells stably transfected with APEX2 constructs were cultured in the same medium with 1 ng/mL puromycin (Life Technologies) after selection.

### Plasmid Transfection.

Transient transfection of HEK293T cells or HeLa cells was performed as previously described (6). Briefly, cells were seeded at 50 to 80% confluence in 6-well plates 24 h prior to transfection. For assays requiring siRNA transfection, transient transfection was performed 32 h after siRNA transfection. Cells were transfected with 1 µg of plasmid DNA using GeneJuice (Merck) following the manufacturer’s protocol. 0.5 µg of each plasmid was used for cotransfection. Assays were performed 40 h after plasmid transfection for siRNA experiments and 24 h posttransfection for other experiments.

### Stable Cell Line Construction.

HeLa cells stably expressing different HLA constructs with APEX2 and FLAG tag linked to the C-terminus were generated by lentiviral transduction. *CLTC and AP2M1* knockout (KO) 293 T cells were established using the CRISPR/Cas9 system (72, 73). Lentivirus was produced as previously described (6). Briefly, lentiviruses were generated in HEK293T cells via cotransfection with 1 μg expression plasmid, 0.5 μg pQ8.91, and 0.25 μg pMD2.G [a gift from Didier Trono (Addgene plasmid # 12259)] in a six-well plate using GeneJuice (Merck). Viral supernatants were collected after 48 h, filtered via a 0.45 µM syringe filter (Fisherbrand), and supplemented with 8 μg/mL polybrene (Santa Cruz Biotechnology). Target cells were seeded at 30% confluency 24 h before transduction and transduced via spinoculation at 800 xg for 30 min at 32 °C. Then, 72 h after transduction, cells were selected with 2 ng/mL puromycin (Life Technologies) for 1 wk. APEX2 expression and KO efficiency were validated by western blot and immunofluorescence.

### Flow Cytometry.

For surface staining, cells were washed twice with Dulbecco’s Phosphate-Buffered Saline (DPBS) (Sigma), stained on ice for 30 min in 50 μL antibody diluted in DPBS, then washed and fixed on ice for 20 min in 100 μL Cytofix (BD Biosciences). For intracellular staining, cells were washed twice with DPBS, fixed, and permeabilized on ice for 20 min in 100 μL Cytofix/Cytoperm (BD Biosciences). After two washes in Perm/Wash buffer (BD Biosciences), cells were stained on ice for 20 min in 50 μL antibody diluted in Perm/Wash buffer, then washed twice more with Perm/Wash buffer, and once with DPBS before resuspension in DPBS. Cells were acquired using an Attune NxT Flow Cytometer (Thermo Fisher Scientific), and data were analyzed using FlowJo 10.4. Gating strategies are shown in *SI Appendix*, Fig.S1. Antibody details and concentrations are listed in *SI Appendix*, Table S4.

### Western Blotting.

Cells were rinsed with DPBS (Sigma) and lysed on ice for 20 min in radioimmunoprecipitation assay (RIPA) buffer [10X RIPA Lysis buffer (Merck) diluted in ddH2O with the addition of a protease inhibitor cocktail (Roche)] (70). Lysates were centrifuged at maximum speed at 4 °C for 15 min to remove cell debris. Equal volumes of lysates were mixed with lithium dodecyl sulfate (LDS) Loading Buffer (Life Technologies), run on 4-12% Bis-Tris gels (Life Technologies) in 3-(N-morpholino) propanesulfonic acid (MOPS) buffer (Life Technologies), and transferred to polyvinylidene fluoride (PVDF) membranes (Merck) using the Trans-Blot Turbo Transfer System (Bio-Rad) in 2X NOVEX Transfer Buffer (Life Technologies). Membranes were blocked for 1 h at room temperature in blocking buffer (5% skim milk, 0.05% Tween-20 in DPBS), followed by a 1-hour incubation with primary antibodies diluted in the wash buffer (DPBS + 0.05% Tween-20). Membranes were washed three times with wash buffer (10 min each), then incubated with secondary antibody diluted in the wash buffer for 1 h at room temperature. After three washings with the wash buffer (10 min each) and a final wash with DPBS, membranes were imaged using a ChemiDoc MP Imaging System (Bio-Rad). Antibody details are provided in *SI Appendix*, Table S4.

### BFA Decay Assay.

BFA decay assay was performed as described previously (6). In brief, cells were treated with brefeldin A (10 µg/mL, BFA, Cambridge Bioscience) for various durations at 37 °C, and surface expression of HLA-E and HLA-A3 constructs was assessed using allophycocyanin (APC) conjugated anti-HLA-E (3D12, BioLegend, 1:200) or anti-GAP.A3 (GAP.A3, Life Technologies, 1:200) antibody. Surface stability was calculated as the percentage of the initial surface expression observed in samples not treated with BFA.

### Internalization Assay.

Internalization assay was adapted from a previously published paper (6). Briefly, HEK293T cells transfected with different constructs were stained on ice for 30 min with APC conjugated anti-HLA-E (3D12, BioLegend, 1:200) or anti-HLA-A3(GAP.A3, Life Technologies, 1:200) antibody, washed with DPBS, and then incubated at 37 °C in prewarmed media with 0.3 mM primaquine (Enzo Life Sciences) for different lengths of time. Cells were resuspended in citric acid buffer (0.1 M, pH 3.0) for 2 min, neutralized with media, washed twice with cold DPBS, and fixed 100 µL of Cytofix (BD Biosciences). The mean fluorescence intensity (MFI) of antibody-labeled cells without acid stripping was set to 100%, and the MFI of antibody-labeled cells with acid stripping (but without internalization) was set to 0%. The percentage of HLA-E internalized after different time points was normalized accordingly.

### Recycling Assay.

The method for the detection and quantification of recycling proteins is adapted from a previously published protocol (24). Where needed, cells were pretreated overnight with NMS-873 (final concentration 5 nM, S7285, Cambridge Bioscience), CB-5083 (final concentration 2 nM, CAY19311, Cambridge Bioscience), or chloroquine (final concentration 20 µM, C6628, Sigma), and the drugs were maintained during the assays. HEK293T cells transfected with different constructs were incubated with unconjugated anti-HLA-E antibody (3D12, BioLegend, 1:200) with or without peptide (50 µM final) diluted in media at 37 °C for 1 h. After washing twice with cold DPBS to remove unbound antibodies, cells were acid-stripped (0.1 M citric acid buffer, pH 3.0) on ice for 2 min to remove uninternalized antibodies, leaving only the intracellular pool of monoclonal antibody (mAb)-bound HLA-E. Following neutralization with 20 volumes of cold media and two washes with cold DPBS, cells were resuspended in prewarmed media with APC-conjugated anti-mouse secondary antibody (Invitrogen, 1:1,000) at 37 °C for varying durations to capture recycled mAb-bound proteins. After two washes with cold DPBS, samples were fixed 100 µL of Cytofix (BD Biosciences) and assessed by flow cytometry. Intracellular mAb-bound HLA-E levels before recycling were measured by intracellular staining as previously described (6). Cells incubated with only the primary or secondary antibody were used as negative controls. The percentage of recycled HLA-E was calculated by normalizing the recycled signal to the total intracellular signal.

### Proximity Labeling and Biotinylated Protein Capture.

Proximity labeling was performed as described previously.(74) Briefly, 10 million HeLa cells stably transfected with various HLA-APEX2-Flag constructs were used for each sample. Biotin-phenol (Sigma, SML2135) was added to the culture medium, and cells were incubated for 30 min. Subsequently, H_2_O_2_ (1 mM final concentration, Sigma) was added for 40 seconds, followed by three washes with quencher buffer (5 mM Trolox (Sigma), 10 mM sodium ascorbate (Sigma), and 10 mM sodium azide (Sigma) in DPBS).

Cells were lysed in lysis buffer (10X RIPA lysis buffer (Merck) diluted in ddH_2_O with cOmplete™ protease inhibitor cocktail (Roche), PhosSTOP™ phosphatase inhibitor (Roche), 5 mM Trolox, 10 mM sodium ascorbate, and 10 mM sodium azide]. Biotinylated proteins were enriched using 100 μL of Pierce™ High Capacity Streptavidin Agarose beads (Thermo), and incubated with rotation for 16 h at 4 °C. Beads were centrifuged at 2,000 ×*g* for 1 min and then sequentially washed twice with 1X RIPA buffer, once with 1X KCl buffer, once with 0.1 M Na_2_CO_3_, once with 2 M urea in 10 mM Tris-HCl (pH 8.0), and finally twice with 1X RIPA buffer. Biotinylated proteins were eluted by boiling the beads in 100 μL of 3X NuPAGE™ LDS Sample Buffer (Thermo) supplemented with 2 mM biotin (Sigma) and 20 mM DTT (Thermo) at 98 °C for 10 min. 5% of the elution was used for western blot as well as for silver staining, and the remaining 90% was reserved for LC–MS preparation. Western blot was performed as described above and silver staining was performed following the manufacturer’s protocol on the eluted samples (Thermo, 24612).

Eluted proteins were processed for mass spectrometry analysis using the S-Trap™ midi digestion protocol (ProtiFi), following the manufacturer’s instructions. Tryptic digest was dried using a speedvac.

### Mass Spectrometry Analysis.

Dried tryptic-digested peptides were reconstituted in 15 µL of LC–MS grade water containing 1% acetonitrile and 0.1% trifluoroacetic acid (TFA). 10% of the sample was analyzed by LC–MS/MS using Dionex Ultimate 3000 Ultra-Performance Liquid Chromatography (UPLC) coupled to a Q-Exactive mass spectrometer (Thermo Fisher Scientific). The peptides were loaded onto a trap column (PepMapC18; 300 µm x 5 mm, 5 µm particle size, Thermo Fisher Scientific) for 1 min at 20 μL/min (30). The loaded peptides were separated on a 50 cm-long chromatographic EasySpray column (ES803, Thermo Fisher Scientific) with a gradient of 2 to 35% acetonitrile in 0.1% formic acid and 5% DMSO at 250 nL/min flow rate for 60 min. Data were acquired using the data-independent acquisition (DIA) mode. Full MS scans were acquired in the Orbitrap mass analyzer over an m/z scan range between 495 and 995 at a resolution of 35 K with an AGC target at 3e6 ions and a maximum injection time of 55 msec. MS2 scans were then acquired over 26 isolation windows of 20 m/z units with a 2 m/z overlap from 495 to 995 m/z. Ions were fragmented in the higher-energy collisional dissociation (HCD) cell with a normalized collision energy of 28%. MS2 spectra were collected in the Orbitrap at a resolution of 17.5 K and automatic gain control (AGC) target of 1e6 ions.

Data were analyzed using DIA-NN 1.8.2 in a library-free mode, employing the UniProt Homo sapiens FASTA database (20,370 entries, retrieved on April 16, 2021). The default search parameters included an allowance for one missed cleavage by trypsin/P, with fixed modifications for N-terminal methionine excision and carbamidomethylation of cysteine residues. The match-between-runs option was enabled for the analysis. The DIA-NN protein groups output matrix was used. All data were imported into Perseus (Version 1.6.15.0), log2 transformed, and filtered to include values present in all replicates for at least one condition. To mitigate potential bias and ensure data integrity, the replicate with the highest missing values for each condition was excluded during quality control. The analysis was conducted with three replicates per condition.

Missing values were imputed using a normal distribution. A two-sample Student’s *t* test in Perseus, with a permutation-based FDR threshold of 5%, was applied to identify significant changes, as shown in the figures. A PCA plot was generated in Perseus using a Benjamini–Hochberg cutoff of 0.05. Volcano and scatter plots were generated in Perseus, and the plots were visualized and prepared in Prism 10.

### Protein–Protein Interaction (PPI) Network Construction.

Proteins showing differential enrichment under either HLA-E or HLA-A3, meeting the filtering criteria (log2[FC] ≥ 1 or ≤ -1, FDR < 0.05), were independently analyzed using the STRING portal. For this analysis, the physical subnetwork type was selected to identify direct PPI from the available databases. Active interaction sources included text mining, experimental data, and curated databases, with a minimum interaction confidence score set at >0.9 (highest-confidence interactions). PPI networks were constructed based on these parameters. PPI networks were constructed based on these parameters using Cytoscape 3.10.1 and were finalized in Adobe Illustrator.

### Immunofluorescence Staining.

Immunofluorescence staining was performed as previously described.(6) Briefly, cells were fixed with 4% formaldehyde (16% formaldehyde (Thermo Fisher Scientific) diluted in DPBS), then permeabilized and blocked in blocking buffer [DPBS containing 0.5% saponin (Merck) and 2% BSA (Merck)]. Anti-RAB7 (Abcam, ab137029) and anti-RAB11 (Life Technologies, 71-5300) primary antibodies were diluted in blocking buffer and applied for 1.5 h, followed by AF568-conjugated secondary antibody (Abcam, ab175471) for 2 h. Cells were mounted with DAPI Fluoromount-G (Cambridge Bioscience), and images were acquired using a Zeiss LSM880 confocal microscope with a 63×/1.4 oil objective.

For the characterization of HeLa cells stably expressing HLA-APEX2-flag constructs, immunofluorescence staining was performed as described previously (74). Briefly, proximity labeling was performed as described above before fixation with 4% paraformaldehyde, followed by permeabilization using 0.2% Triton-X (Sigma, T9284) in DPBS for 10 min at 25 °C. After blocking with 1% BSA in DPBS for 1 to 2 h at 25 °C, cells were incubated with anti-flag antibody (Sigma, F1804) and IRDye 800CW Streptavidin antibody (LI-COR, 926-32230) overnight at 4 °C, followed by incubation with AF647-conjugated goat anti-mouse secondary antibody (Invitrogen, A32728) at RT for 1 h. The plate was imaged on a high-content laser-based spinning disk confocal microscope (Opera Phenix Plus, Revvity), using a 60× water objective.

Image analysis, including colocalization quantification, was performed in Fiji/ImageJ using the Coloc2 plug-in. Antibody details and concentrations are listed in *SI Appendix*, Table S4.

### siRNA Transfection.

To identify host factors involved in regulating the endosomal trafficking of HLA-E, an RNAi-based screen was performed using a commercially available library targeting 140 cellular membrane-trafficking genes (Dharmacon, GU-105500-01). THP-1 cells were plated in 96-well plates for surface expression screening while HEK293T cells were plated in 24-well plates for internalization and recycling assay. siRNA transfection was performed using RNAiMAX (Thermo) according to the manufacturer’s instructions using reverse transfection at a final concentration of 10 nM. Plasmid transfection was performed 32 h after plasmid transfection, and assays were performed 72 h after transfection.

### ELISA-Based Peptide Binding Assay.

CCVs were purified from 80 g frozen porcine brain tissue, and 2 mL of CCV was obtained as previously described (75, 76). CCV coat proteins (a mixture of clathrin and adaptors) were then extracted using 500 μL of extraction buffer [2.5 M Tris, pH 7.3, 200 mM EDTA (Invitrogen), 1 mM β-mercaptoethanol (Sigma), protease inhibitor (Roche)]. Coat protein extract was diluted 1:4 in binding buffer (0.5 M Tris, pH 7.3, 2 mM EDTA) for binding to biotinylated peptides. N-terminal biotin-labeled peptides (*SI Appendix*, Table S1) were diluted to 5 μg/mL in DPBS, followed by 50 μL of this dilution was immobilized onto streptavidin-coated plates (RandD Systems, CP003) for 1 h at RT. After washing five times with DPBS (300 μL/each), plates were blocked with 300 μL blocking buffer (2% IgG-free bovine serum albumin (Gibco) in DPBS) for 2 h at RT. After washing five times with DPBS (300 μL/each), 50 μL AP-2 enriched samples were added to each well and incubated at 4 °C overnight. After washing five times with blocking buffer, 50 μL anti-AP2A1 antibody AP.6 [produced in the lab as previously described (77)] diluted to 5 μg/mL in blocking buffer was added and incubated at 4 °C for 2.5 h. After washing five times with blocking buffer, 50 μL horseradish peroxidase (HRP)-linked anti-mouse antibody (Cell Signaling, 7076) diluted in blocking buffer (1:2,000) was added and incubated at 4 °C for 1.5 h. After washing five times with blocking buffer and twice with DPBS, tetramethyl benzidine substrate and STOP solution were used to develop and terminate reactions, respectively, following the manufacturer’s protocol (Biolegend). Absorbance readings were obtained at 450 nm on a FLUOstar OMEGA plate reader.

### Statistical Analysis.

At least three independent replicates were performed for all experiments. Summary graphs and error bars for ELISA represent means ± SEM, and for other data represent means± SD. Ordinary one-way ANOVA (ANOVA) with Tukey’s post hoc test, paired two-tailed *t* test with Welch’s correction, or one-sample *t* test were used for comparisons and were performed using Prism 10. All comparisons and tests are noted in the figure legends in which they appear.

## Supplementary Material

Appendix 01 (PDF)

Dataset S01 (PDF)

Dataset S02 (XLSX)

Dataset S03 (XLSX)

## Data Availability

The mass spectrometry proteomics data have been deposited in the ProteomeXchange Consortium via the PRIDE partner repository (78) with the dataset identifier PXD060886 and are publicly available as of the date of publication (78). Original western blot images for Fig. 6 are shown in Data file S1 and data for plotting the graphs in Figs. 4 and 5 are available in data files S2 and S3 respectively. All study data are included in the article and/or supporting information.
