# Supplementary material for: A cytoplasmic motif in HLA-E that drives clathrin-mediated endocytosis and VCP-associated postendocytic trafficking
Source: Proc Natl Acad Sci U S A. 2025 Oct 24;122(43):e2514956122. doi: 10.1073/pnas.2514956122 (PMC12582296; doi:10.1073/pnas.2514956122)
Supplement: Supplementary file 1 — Appendix 01 (PDF) [file pnas.2514956122.sapp.pdf]

PNAS

Supporting Information for

A cytoplasmic motif in HLA-E that drives clathrin-mediated endocytosis and VCP-associated post-endocytic trafficking

Wanlin He[1,2], Andreas Damianou[3,4], Iolanda Vendrell[3], Klaus Früh[5], Daowen I. Yin[6,7], Frances M. Brodsky[6,7], Benedikt M. Kessler[3,4], Simon Brackenridge[1], Persephone Borrow[1], Geraldine M. Gillespie[1], Andrew J. McMichael[1,4,*]

[1]Center for Immuno-Oncology, Nuffield Department of Medicine, University of Oxford, Roosevelt Drive, Oxford, OX3 7DQ, UK

[2]State Key Laboratory of Oral Diseases & National Center for Stomatology and National Clinical Research Center for Oral Diseases, West China Hospital of Stomatology, Sichuan University, Chengdu, 610041, China

[3]Target Discovery Institute, Centre for Medicines Discovery, Nuffield Department of Medicine, University of Oxford, Roosevelt Drive, Oxford, OX3 7FZ, UK

[4]Chinese Academy of Medical Sciences Oxford Institute, Nuffield Department of Medicine, University of Oxford, Roosevelt Drive, Oxford, OX3 7BN, UK

[5]Vaccine and Gene Therapy Institute and Oregon National Primate Research Center, Oregon Health and Science University, Beaverton, OR 97006, USA

[6]Department of Structural and Molecular Biology, Division of Biosciences, University College London, Gower Street, London, WC1E 6BT, UK

[7]Institute of Structural and Molecular Biology, Birkbeck and University College London, London, WC1E 7HX, UK

***Correspondence:**

Andrew J. McMichael, E-mail: andrew.mcmichael@ndm.ox.ac.uk

This PDF file includes:

Figures S1 to S12

Tables S1 to S4

Other supporting materials for this manuscript include the following:

Datasets S1 to S6

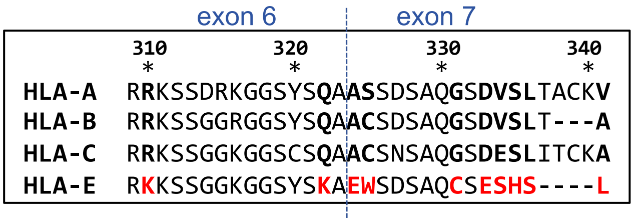

Fig. S1. Sequences of the cytoplasmic tail of different HLA-I molecules
HLA-E-unique amino acids are highlighted in red.

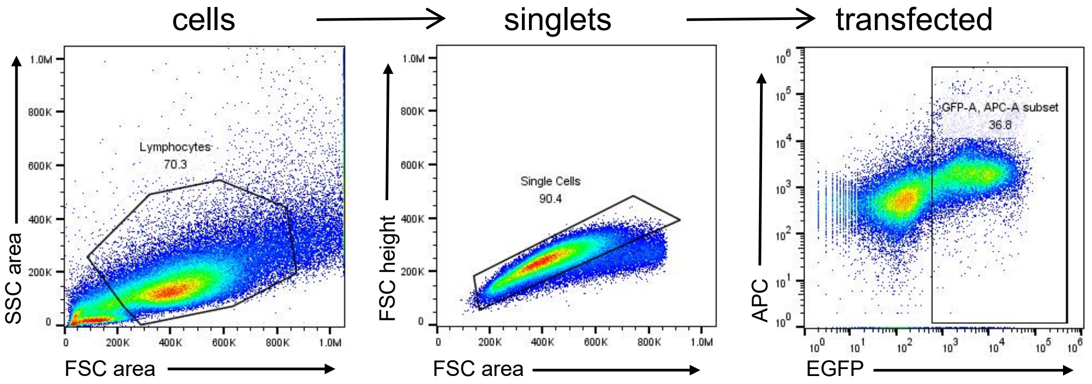

Fig. S2. Representative example of gating strategy

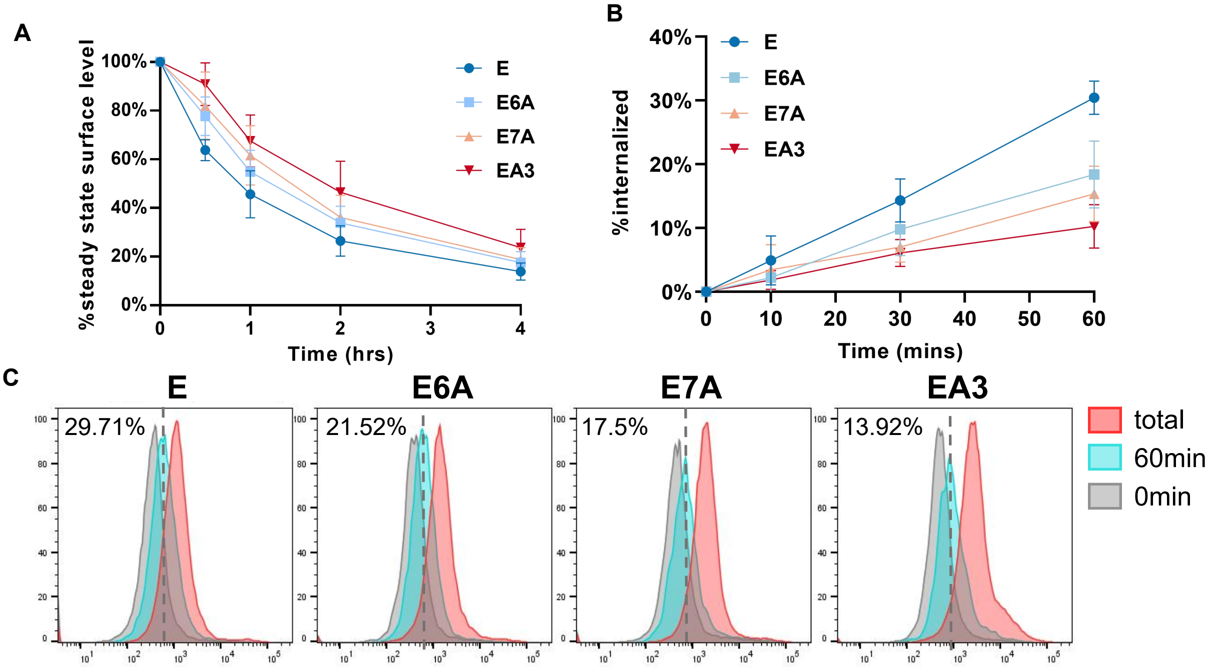

Fig. S3. Both exon 6 and exon 7 are required to promote HLA-E internalization

(A) BFA assay in HEK293T cells transiently transfected with different HLA-E constructs shown in Figure 1A. The average cell surface MFI before BFA addition was set to 100%, and the MFI after BFA incubation at various time points was normalized to this percentage.

(B) Internalization assay in HEK 293T cells transiently transfected with different HLA-E constructs from Figure 1A. The MFI of antibody-labeled cells without acid stripping was set to 100%, and the MFI of antibody-labeled cells with acid stripping (but without internalization) was set to 0%. The percentage of HLA-E internalized after different time periods was normalized accordingly.

(C) Representative stainings for 1-hour internalization (60min, blue) from Figure 1E. The MFI of antibody-labeled cells without acid stripping was set to 100% (total, red), and the MFI of antibody-labeled cells with acid stripping (but without internalization) was set to 0% (0min, gray). The median MFI for 1-hour internalization samples is indicated with a dashed line, and the percentage of internalized HLA-E is shown in the upper left corner. Data were collected from six (A, B) replicates and are presented as mean ± SD (error bars).

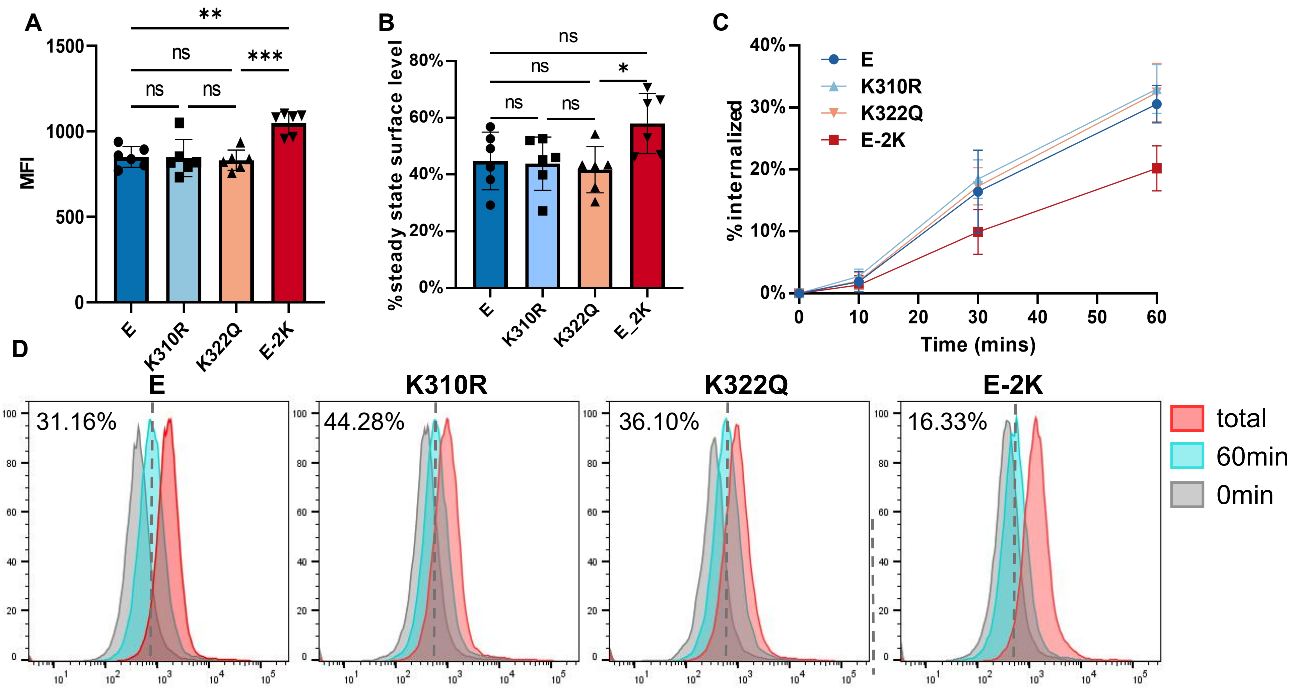

Fig. S4. Two unique lysines on exon6 faciliates HLA-E internalization

(A) Flow cytometry analysis of HEK293T cells transiently transfected with different HLA-E constructs shown in Figure 1F.

(B) BFA assay in HEK293T cells transiently transfected with different HLA-E constructs shown in Figure 1F. The average cell surface MFI before BFA addition was set to 100%, and the MFI after BFA incubation for 1 hour was normalized as its percentage. Data were collected for six replicates and are shown as mean ± SD (error bars).

(C) Internalization assay in HEK 293T cells transiently transfected with different HLA-E constructs from Figure 1F. The MFI of antibody-labeled cells without acid stripping was set to 100%, and the MFI of antibody-labeled cells with acid stripping (but without internalization) was set to 0%. The percentage of HLA-E internalized after different time periods was normalized accordingly.

(D) Representative stainings for 1-hour internalization (60min, blue) from Figure 1G. The MFI of antibody-labeled cells without acid stripping was set to 100% (total, red), and the MFI of antibody-labeled cells with acid stripping (but without internalization) was set to 0% (0min, gray). The median MFI for 1-hour internalization samples is indicated with a dashed line, and the percentage of internalized HLA-E is shown in the upper left corner. Data were collected for five (C) or six (A, B) replicates and are shown as mean ± SD (error bars). Statistical analysis was performed using one-way ANOVA with Tukey's post-hoc test. Asterisks show the statistical significance between indicated groups: ns, not significant; *, $P < 0.05$; **, $P < 0.01$; ***, $P < 0.001$.

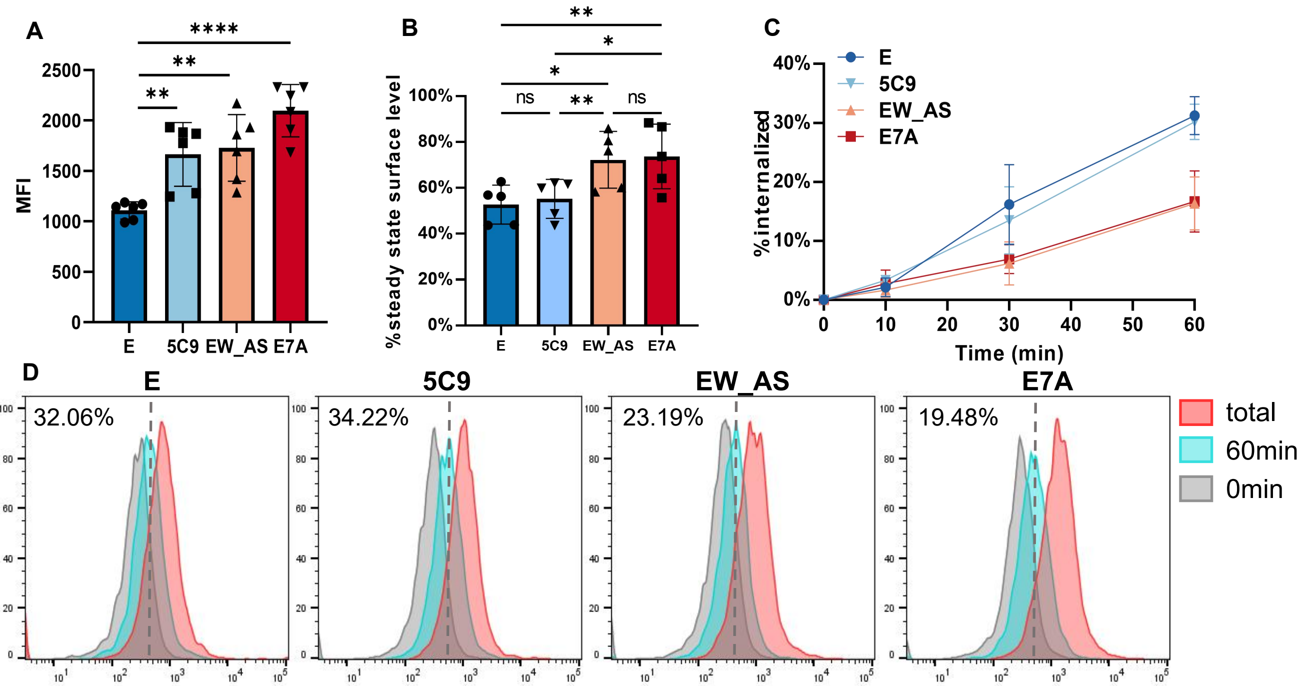

Fig. S5. Internalization-promoting effect of exon7 depends on the first two amino acids

(A) Flow cytometry analysis of HEK293T cells transiently transfected with different HLA-E constructs shown in Figure 1F.

(B) BFA assay in HEK293T cells transiently transfected with different HLA-E constructs shown in Figure 1F. The average cell surface MFI before BFA addition was set to 100%, and the MFI after BFA incubation for 1 hour was normalized as its percentage. Data were collected for six replicates and are shown as mean ± SD (error bars).

(C) Internalization assay in HEK 293T cells transiently transfected with different HLA-E constructs from Figure 1F. The MFI of antibody-labeled cells without acid stripping was set to 100%, and the MFI of antibody-labeled cells with acid stripping (but without internalization) was set to 0%. The percentage of HLA-E internalized after different time periods was normalized accordingly.

(D) Representative stainings for 1-hour internalization (60min, blue) from Figure 1H. The MFI of antibody-labeled cells without acid stripping was set to 100% (total, red), and the MFI of antibody-labeled cells with acid stripping (but without internalization) was set to 0% (0min, gray). The median MFI for 1-hour internalization samples is indicated with a dashed line, and the percentage of internalized HLA-E is shown in the upper left corner. Data were collected for five (C) or six (A, B) replicates and are shown as mean ± SD (error bars). Statistical analysis was performed using one-way ANOVA with Tukey's post-hoc test. Asterisks show the statistical significance between indicated groups: ns, not significant; *, $P < 0.05$; **, $P < 0.01$; ***, $P < 0.001$.

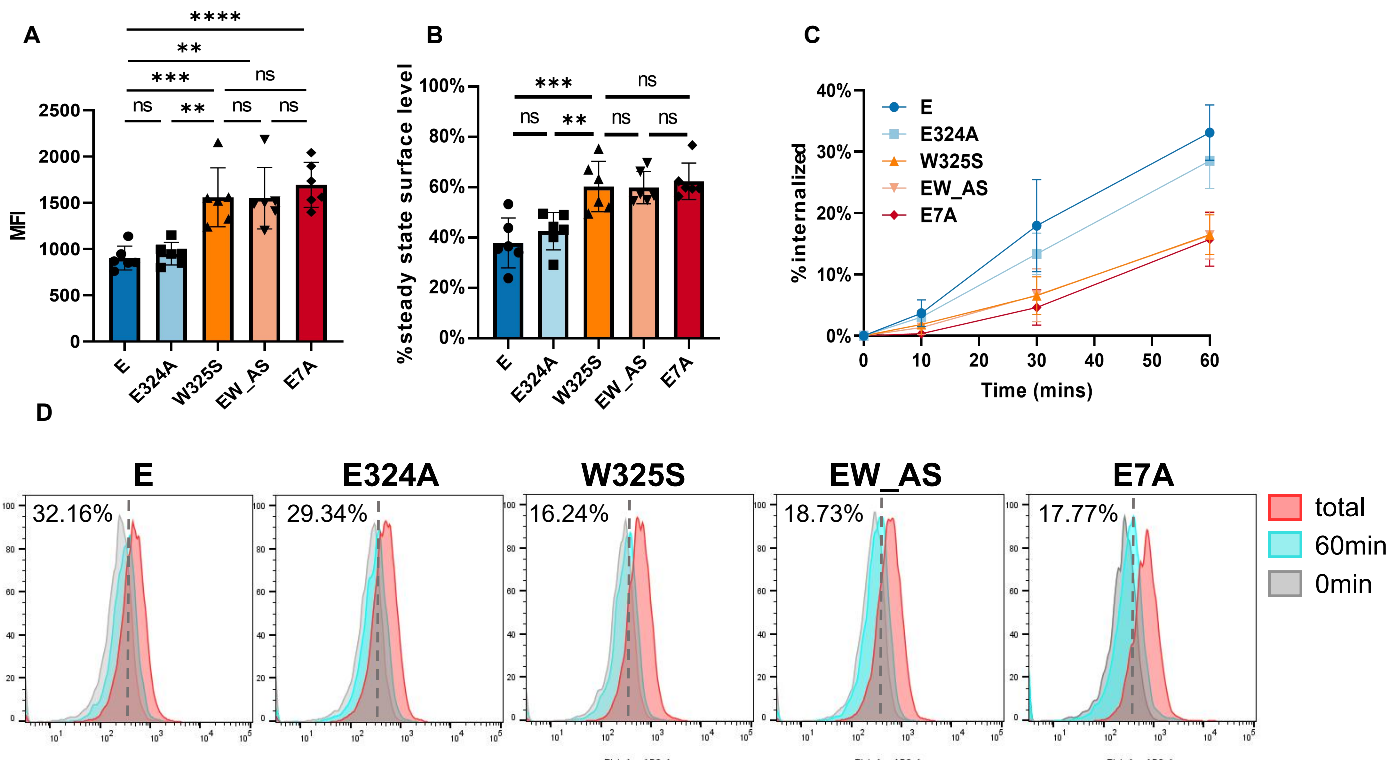

Fig. S6. A unique tryptophan in exon 7 contributes to HLA-E internalization

(A) Flow cytometry analysis of HEK293T cells transiently transfected with different HLA-E constructs shown in Figure 1F.

(B) BFA assay in HEK293T cells transiently transfected with different HLA-E constructs shown in Figure 1F. The average cell surface MFI before BFA addition was set to 100%, and the MFI after BFA incubation for 1 hour was normalized as its percentage. Data were collected for six replicates and are shown as mean ± SD (error bars).

(C) Internalization assay in HEK 293T cells transiently transfected with different HLA-E constructs from Figure 1F. The MFI of antibody-labeled cells without acid stripping was set to 100%, and the MFI of antibody-labeled cells with acid stripping (but without internalization) was set to 0%. The percentage of HLA-E internalized after different time periods was normalized accordingly.

(D) Representative stainings for 1-hour internalization (60min, blue) from Figure 1I. The MFI of antibody-labeled cells without acid stripping was set to 100% (total, red), and the MFI of antibody-labeled cells with acid stripping (but without internalization) was set to 0% (0min, gray). The median MFI for 1-hour internalization samples is indicated with a dashed line, and the percentage of internalized HLA-E is shown in the upper left corner. Data were collected for six replicates (A-C) and are shown as mean ± SD (error bars). Statistical analysis was performed using one-way ANOVA with Tukey's post-hoc test. Asterisks show the statistical significance between indicated groups: ns, not significant; *, $P < 0.05$; **, $P < 0.01$; ***, $P < 0.001$.

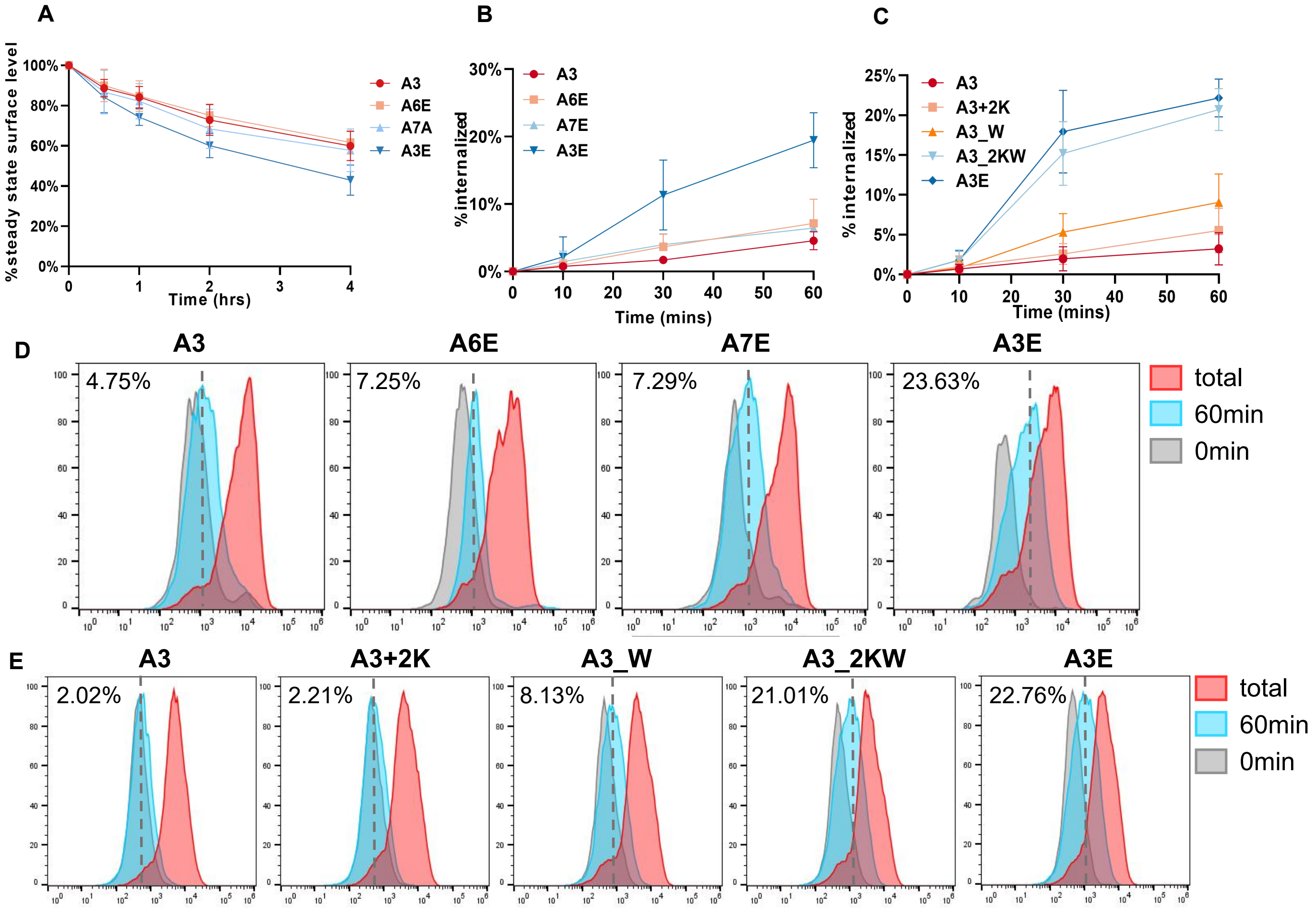

Fig. S7. The lysine/tryptophan-based motif is sufficient to promote fast internalization of HLA-I proteins

The average cell surface MFI before BFA addition was set to 100%, and the MFI after BFA incubation for 4hrs was normalized as its percentage. Data were collected for six replicates and are shown as mean ± SD (error bars).

(B, C) Internalization assay in HEK 293T cells transiently transfected with different HLA-A3 constructs from Figure 2A (B) and Figure 2E (C). The MFI of antibody-labeled cells without acid stripping was set to 100%, and the MFI of antibody-labeled cells with acid stripping (but without internalization) was set to 0%. The percentage of HLA-A3 internalized after different time periods was normalized accordingly.

(D, E) Representative stainings for 1-hour internalization (60min, blue) from Figure 2D (D) and Figure 2G (E). The MFI of antibody-labeled cells without acid stripping was set to 100% (total, red), and the MFI of antibody-labeled cells with acid stripping (but without internalization) was set to 0% (0min, gray). The median MFI for 1-hour internalization samples is indicated with a dashed line, and the percentage of internalized HLA-E is shown in the upper left corner. Data were collected for six replicates (A-C) and are shown as mean ± SD (error bars).

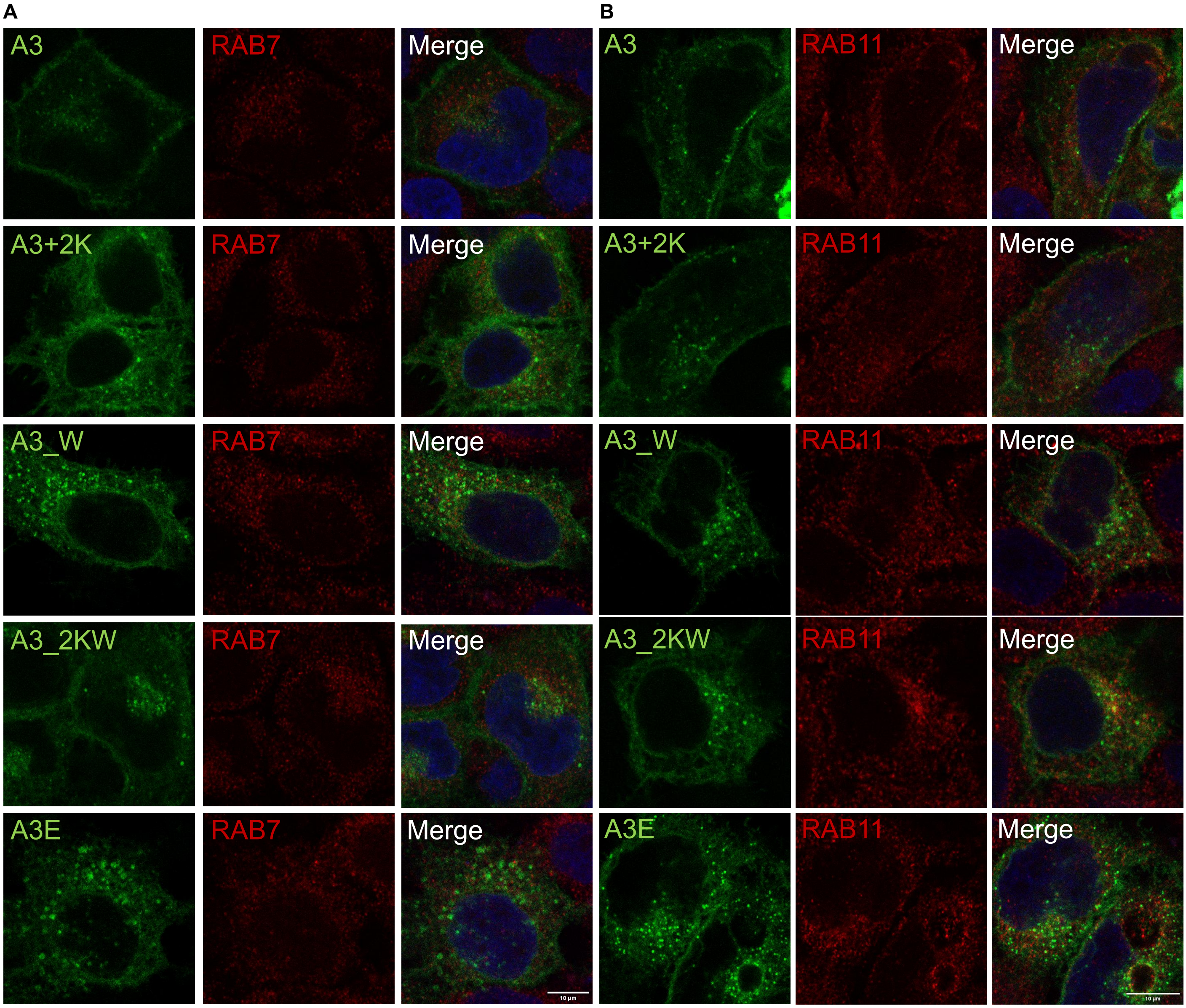
A
A3
RAB7
Merge
A3+2K
RAB7
Merge
A3_W
RAB7
Merge
A3_2KW
RAB7
Merge
A3E
RAB7
Merge
B
A3
RAB11
Merge
A3+2K
RAB11
Merge
A3_W
RAB11
Merge
A3_2KW
RAB11
Merge
A3E
RAB11
Merge

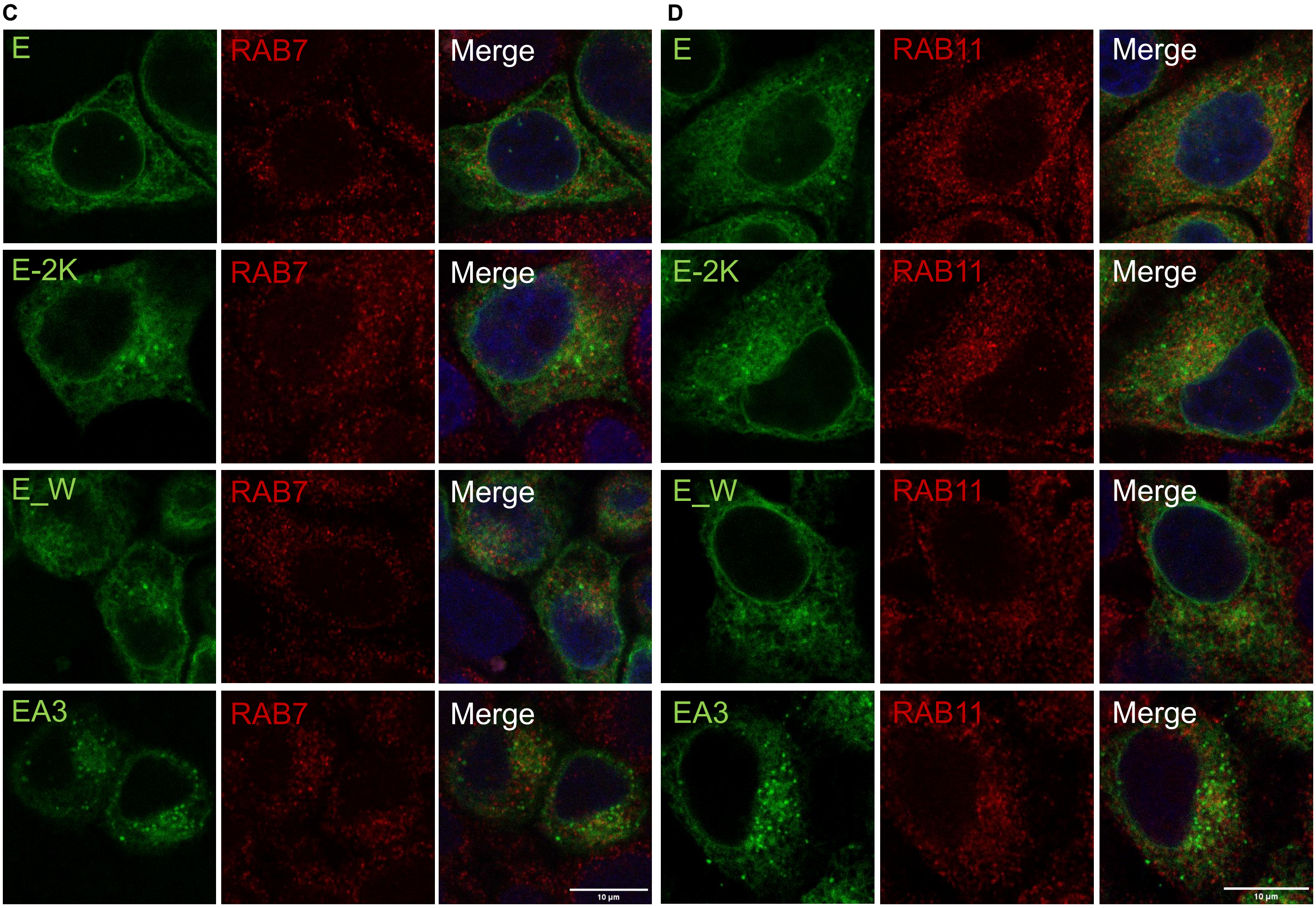

Fig.S8 Co-localization of different HLA constructs with endosomal markers
Representative confocal micrographs of HeLa cell transfected with different HLA-A3 (**A, B**) or HLA-E (**C, D**) constructs in Fig.2H-K. Cells were fixed, permeabilized, and stained with antibodies against protein markers for late eondosome (RAB7; **A, C**) or recycling endosome (RAB11; **B, D**), followed by detection with an Alexa568-conjugated secondary antibody. Scale bars = 10 µm.

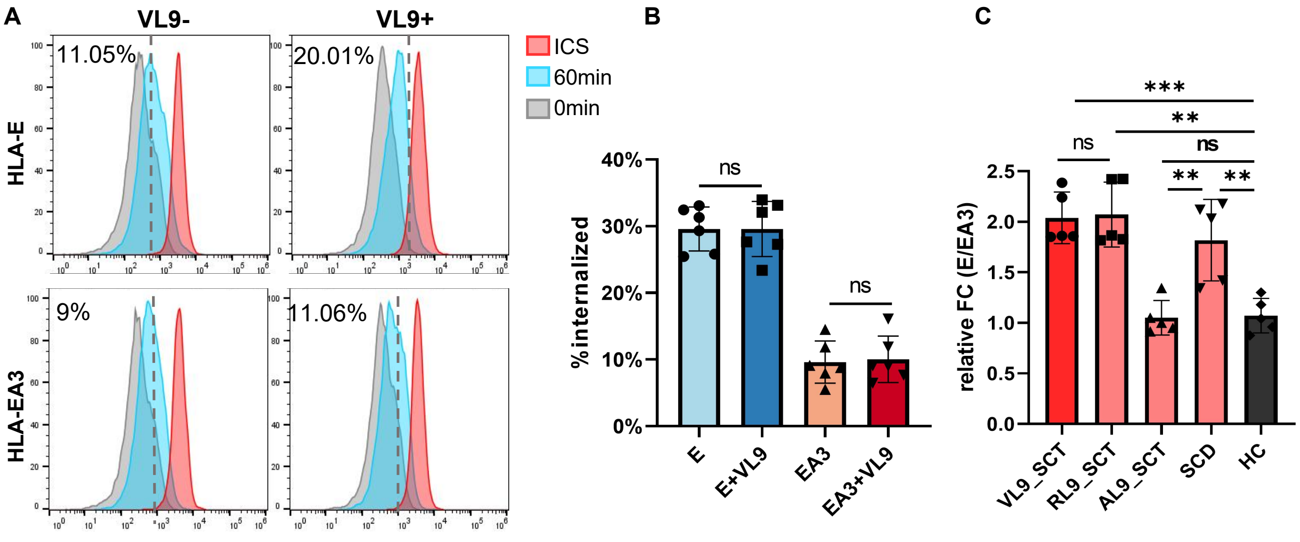

Fig. S9. Strong binding peptides facilitate HLA-E surface reappeareance

(A) Representative stainings for 1-hour recycling (60min, blue) from Figure 3B. The total internalized mAb-bound HLA-E after acid stripping was assessed by intracellular staing and was set to 100% (ICS, red). The MFI of antibody-labeled cells with acid stripping (but without recycling) was set to 0% (0min, gray). The percentage of HLA-E recycled after 1h was normalized accordingly. The median MFI for 1-hour internalization samples is indicated with a dashed line, and the percentage of internalized HLA-E is shown in the upper left corner.

(B) Internalization assay in HEK 293T cells transiently transfected with different HLA-E constructs in (A) with or without VL9 peptide pulsing during the internalization stage. The MFI of antibody-labeled cells without acid stripping was set to 100%, and the MFI of antibody-labeled cells with acid stripping (but without internalization) was set to 0%. The percentage of HLA-E internalized after 1h was normalized accordingly. Data were collected for six replicates and are shown as mean ± SD (error bars). Statistical analysis was performed using paired two-tailed t-tests with Welch's correction. ns: not significant.

(C) Recycling assay in HEK 293T cells transiently transfected with different SCTs, SCD, or HC of HLA-E or HLA-EA3. Recycling assay was carried out as described for Figure 3A. The recycling-promoting effect was assessed by calculating the FC increase of the percentage of HLA-E recyced with VL9 pusling to the percentage without VL9. The FC of HLA-E constructs was normalized to the corresponding HLA-EA3 constructs (relative FC). Data were collected for five biological runs and are shown as mean ± SD (error bars). Statistical analysis was performed using one-way ANOVA with Tukey's post-hoc test. Asterisks show the statistical significance between indicated groups: ns, not significant;**, P < 0.01; ***, P < 0.001.

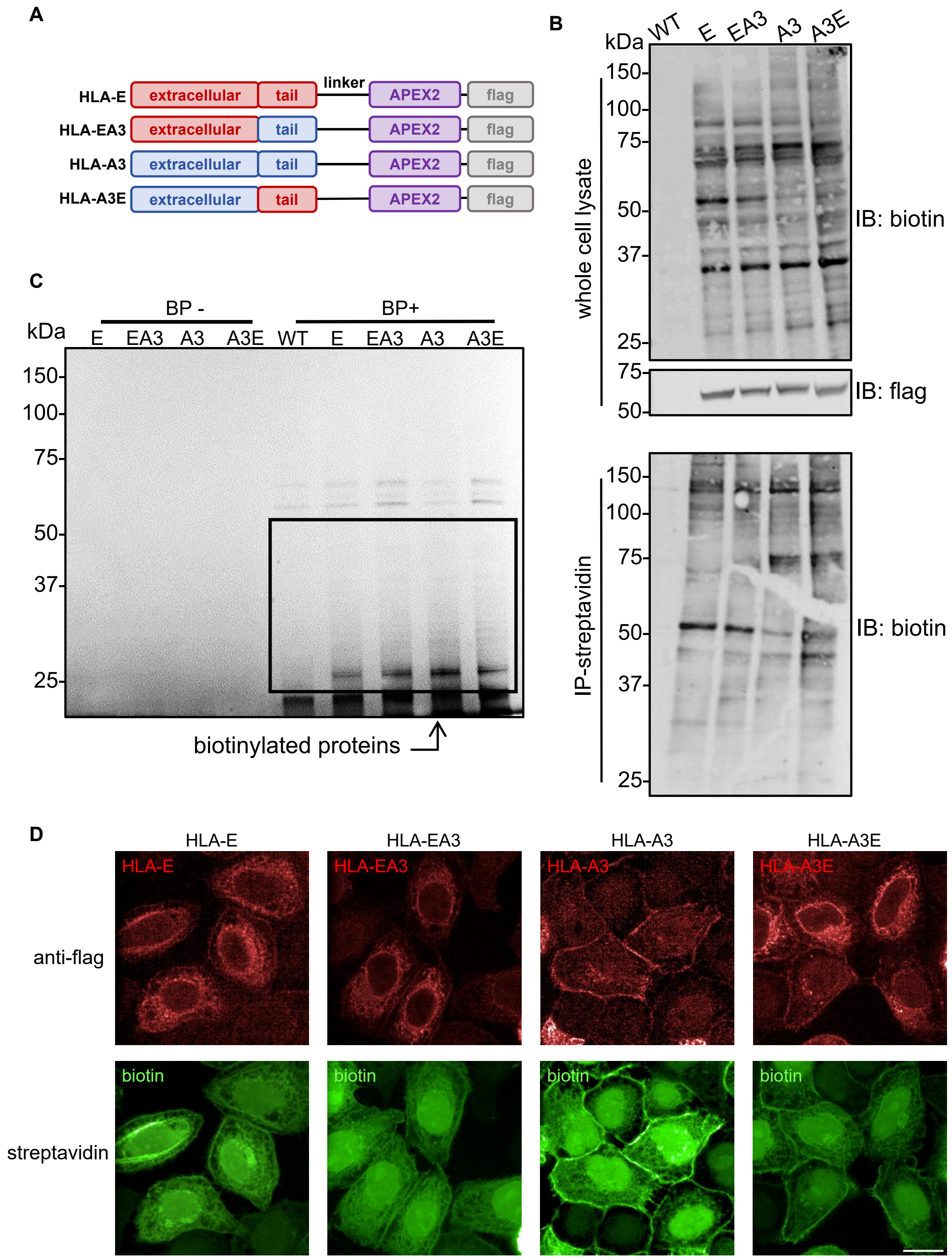

Fig. S10. HLA-APEX2-fused constructs enable efficient labeling and recapitulate intracellular and membrane localization
(A) Schematic representation of APEX2 tagging HLA constructs. APEX2 was fused to the C terminus of different HLA constructs via a glycine-serine linker, followed by a FLAG tag.
(B) Immunoblots of whole-cell lysate and streptavidin pull-down (IP) biotinylated proteins in HeLa cells stably transfected with different HLA-APEX2 constructs after APEX2 proximity labeling. HLA-APEX2 or biotinylated proteins were detected with anti-flag or IRDye 800CW streptavidin antibody, respectively.
(C) Silver staining of streptavidin-enriched biotinylated proteomes as described in B.
(D) Confocal fluorescence imaging of HLA-APEX2 labeling in HeLa cell lines. HeLa cells stably expressing HLA-E, HLA-EA3, HLA-A3, HLA-A3E tagged with APEX2 and flag on the C-terminus were incubated with BP, followed by H_2O_2. Afterward, cells were fixed and stained with anti-flag antibody to visualize the localization of different HLA-APEX2 and streptavidin conjugated to Alexa Fluor 488 to visualize biotinylated proteins. Scale bars = 10 μm.

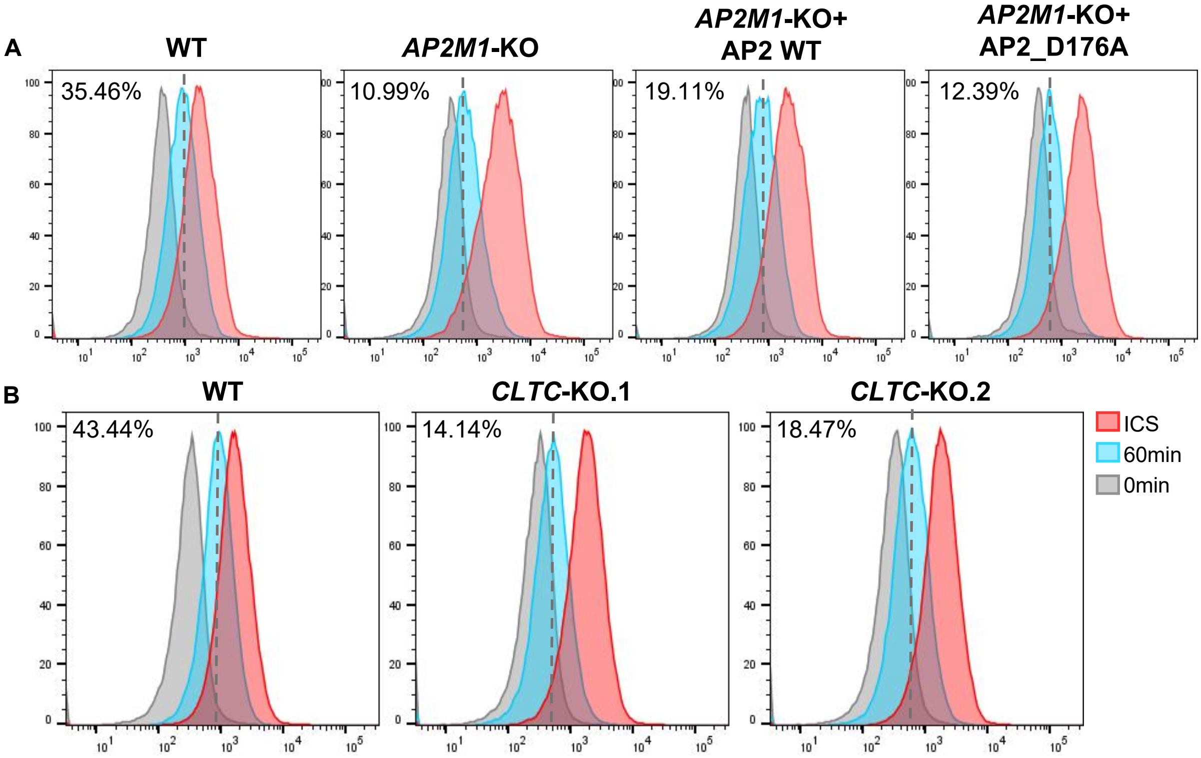

Fig. S11. HLA-E internalization depends on AP-2 complex and clathrin
Representative stainings for 1-hour internalization (60min, blue) of HLA-E from Figure 6B (A) and Figure 6D (B). The MFI of antibody-labeled cells without acid stripping was set to 100% (total, red), and the MFI of antibody-labeled cells with acid stripping (but without internalization) was set to 0% (0min, gray). The median MFI for 1-hour internalization samples is indicated with a dashed line, and the percentage of internalized HLA-E is shown in the upper left corner.

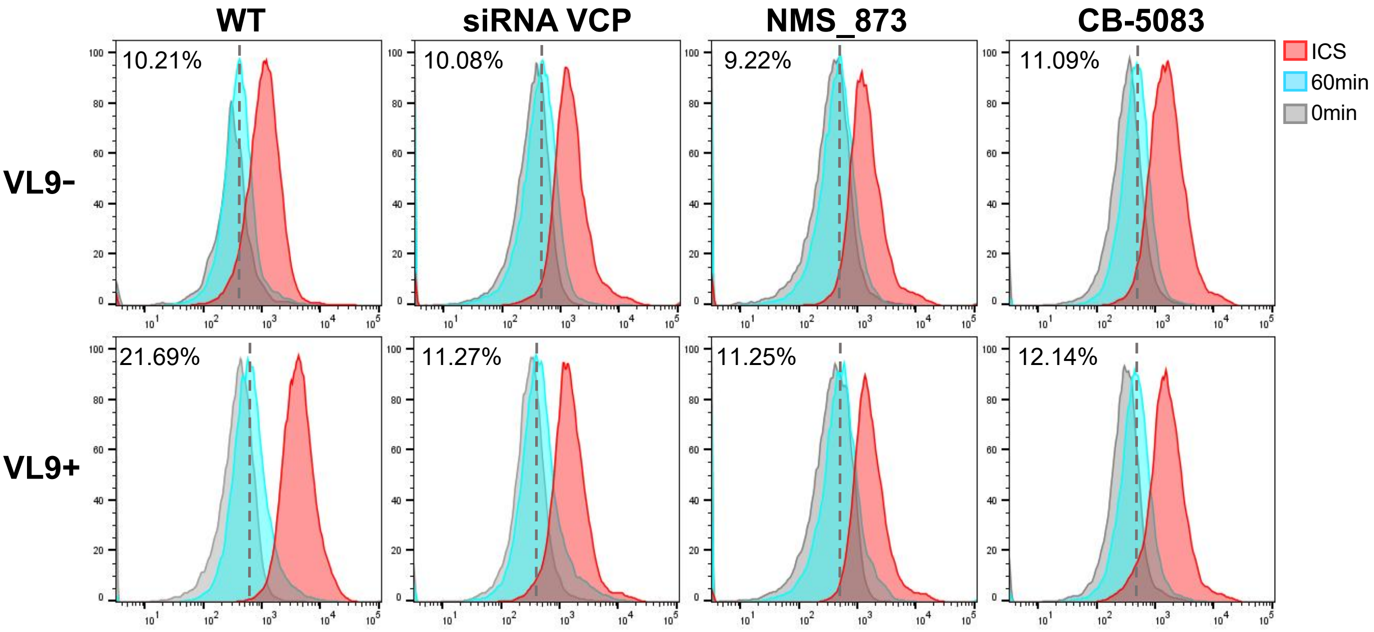

Fig. S12. HLA-E cytoplasmic tail and strong binding peptides promote its surface reappearance via a VCP-dependent pathway

Representative stainings for 1-hour recycling (60min, blue) from Figure 6H. The total internalized mAb-bound HLA-E after acid stripping was assessed by intracellular staing and was set to 100% (ICS, red). The MFI of antibody-labeled cells with acid stripping (but without recycling) was set to 0% (0min, gray). The percentage of HLA-E recycled after 1h was normalized accordingly. The median MFI for 1-hour internalization samples is indicated with a dashed line, and the percentage of internalized HLA-E is shown in the upper left corner.

Table S1 Peptide sequences

peptide	sequence
VL9	VMAPRTVLL
Mtb44	RLPAKAPLL
RL9	RMYSPTSIL
AL9	ATPLLMQAL
E tail	biotin-GSGSGSGSGRKKSSGGKGGSYSKAEWSDSAQCSESHSL
E_mut tail	biotin-GSGSGSGSGRRKSSGGKGGSYSQAESSDSAQCSESHSL
CD1 tail	biotin-GSGSGRRRSYQNIP
CD1_mut tail	biotin-GSGSGRRRSAQNIP

Table S2 Primers for plasmid construction

primer	sequence
pGEN F	CTACCGGACTCAGATCTCGAGCTCA
PGEN TER R	GGTATGGCTGATTATGATCTAGAGTCGC
E7A F	GGAGCTACTCTAAGGCTGAGAGCAGTGACAGTGCCCAGG
E7A R	CCTGGGCACTGTCACTGCTCTCAGCCTTAGAGTAGCTCC
A7E F	GGAGTTACACTCAGGCTGCATGGAGCGACAGTGCCCAGG
A7E R	CCTGGGCACTGTCGCTCCATGCAGCCTGAGTGTAACTCC
K310R F	CTGTGATATGGAGGAGGAAGAGCTCAGGTGG
K310R R	CCACCTGAGCTCTTCCTCCTCCATATCACAG
K322Q F	AGGGAGCTACTCTCAGGCTGAGTGGAGC
K322Q R	GCTCCACTCAGCcTGAGAGTAGCTCCCT
A3+2K F	GCTCAGATAGAAAAGGAGGGAGTTACACTAAGGCTGCAAGCAGTGA
A3+2K R	AACTCCCTCCTTTTCTATCTGAGCTCTTCTTCCTCCACATCACGG
5C9 F	TGGAGCGACAGTGCCCAGGGCTCTGATG
5C9 R	AGCCCTGGGCACTGTCGCTCCA
EW-AS F	CTCTAAGGCTGCAAGCAGCGACAGTGCCCAGG
EW-AS R	CACTGTCGCTGCTTGCAGCCTTAGAGTAGCTC
E324A F	CTCTAAGGCTGCATGGAGCGACAGTGCCCAGG
E324A R	CACTGTCGCTCCATGCAGCCTTAGAGTAGCTC
W325S F	CTCTAAGGCTGAGAGCAGCGACAGTGCCCAGG
W325S R	CACTGTCGCTGCTCTCAGCCTTAGAGTAGCTC
A3_W F	AGGCTGCATGGAGTGACAGTGCCCAGGGCTCTG
A3_W R	CCTGGGCACTGTCACTCCATGCAGCCTGAGTGTAACTCC
A3_2KW R	CCTGGGCACTGTCACTCCATGCAGCCTTAGTGTAACTCC
MamuCTD_F	TGGTTGCTGCTGTGATATGGAGGAGGAAGAGCTCAGGTAGAA
MamuCTD_R	TTCTACCTGAGCTCTTCCTCCTCCATATCACAGCAGCAACCA
Mamu-E0204_F	TAGAAAAGGAGGGAGCTACTCTCAGGCTGTGGGT
Mamu-E02_R	GAGAGTAGCTCCCTCCTTTTCTACCTGAGCTCTTCCTCCT
Mamu-E0216_F	TAGAAAAGGAGGGAGCTACTCTCAGGCTTCGTGTAGC
Mamu-E0201_F	CACAGCTTGTAAAGCGGATCCACCGGTCGCCA
Mamu-E0201_R	GTGGATCCGCTTTACAAGCTGTGAGAGACTCATCAGA
SN_F	ATATGGAGGTCGAACAGCTCAGGTAGAAAAGGATGGAG
SN_R	ACCTGAGCTGTTCGACCTCCATATCACAGCAGCAAC
MamuE0221_F	CTGTGTGTAGCGACAGTGCCCAGGGATCTGATGAGTCTC
MamuE0221_R	TGGGCACTGTCGCTACACACAGCCTGAGAGTAGCTCC
MamuE0221_F2	TAGAAAAGGAGGGAGCTACTCTCAGGCTGTG
MamuE0221_R2	GAGAGTAGCTCCCTCCTTTTCTACCTGAGCTGTTC
E_BamHI_F	AGAAGACACCGACTCTAGAGGATCCGCCACCATGGTAGATGGAACCCTCCT
A3_BamHI_F	AGAAGACACCGACTCTAGAGGATCCGCCACCATGGCCGTCATGGC
E_APEX2_F	CCAGGGGTCTGAGTCTCACAGCTTGGGAAGTGGAAGTGGAAGTGGAGGAAAGT
E_APEX2_R	ACTTTCCTCCACTTCCACTTCCACTTCCCAAGCTGTGAGACTCAGACCCCTGG
A3_APEX2_F	TGATGTGTCCCTCACAGCTTGTAAAGTGGGAAGTGGAAGTGGAAGTGGAGGAAAGT
A3_APEX2_R	ACTTTCCTCCACTTCCACTTCCACTTCCCACTTTACAAGCTGTGAGGGACACATCA
APEX2_flag_SalI_R	TGTAATCCAGAGGTTGATTGTCGACTCACTTATCGTCGTCATCCTTGTAGTCGGCATC AGCAAACCCAAGCTCG

Table S3 Strategies for constructs generation

Construct	Part 1 primer	Part 2 primer	Part 1 backbone	Part 2 backbone
HLA-E6A_EGFP	A7E R	A7E F	HLA-EA3_EGFP	HLA-E_EGFP
HLA-E7A_EGFP	E7A R	E7A F	HLA-E_EGFP	HLA-A3_EGFP
HLA-A6E_EGFP	E7A R	E7A F	HLA-A3E_EGFP	HLA-A3_EGFP
HLA-A7E_EGFP	A7E R	A7E F	HLA-A3_EGFP	HLA-E_EGFP
HLA-E_K310R_EGFP	K310R R	K310R F	HLA-E_EGFP	HLA-E_EGFP
HLA-E_K322Q_EGFP	K322Q R	K322Q F	HLA-E_EGFP	HLA-E_EGFP
HLA-E-2K_EGFP	K322Q R	K322Q F	HLA-E_K310R_EGFP	HLA-E_K310R_EGFP
HLA-A3+2K_EGFP	A3+2K R	A3+2K F	HLA-A3_EGFP	HLA-A3_EGFP
HLA-E_5C9_EGFP	5C9 R	5C9 F	HLA-E_EGFP	HLA-A3_EGFP
HLA-E_EW_AS_EGFP	EW_AS R	EW_AS F	HLA-E_EGFP	HLA-E_EGFP
HLA-E_E324A_EGFP	E324A R	E324A F	HLA-E_EGFP	HLA-E_EGFP
HLA-E_W325S_EGFP	W325S R	W325S F	HLA-E_EGFP	HLA-E_EGFP
HLA-A3_W_EGFP	A3_W R	A3_W F	HLA-A3_EGFP	HLA-A3_EGFP
HLA-A3_2KW_EGFP	A3_2KW R	A3_W F	HLA-A3+2K_EGFP	HLA-A3+2K_EGFP
HLA-E_mamu0204_EGFP	MamuCTD_R	MamuCTD_F	HLA-E_EGFP	SCT_Mamu0204_EGFP
HLA-E_mamu0216_EGFP	MamuCTD_R	MamuCTD_F	HLA-E_EGFP	SCT_Mamu0216_EGFP
HLA-E_mamu0204W->G_EGFP	Mamu-E02_R	Mamu-E0204_F	HLA-E_mamu0204_EGFP	HLA-E_mamu0204_EGFP
HLA-E_mamu0216W->G_EGFP	Mamu-E02_R	Mamu-E0216_F	HLA-E_mamu0216_EGFP	HLA-E_mamu0216_EGFP
HLA-E_mamu0201_EGFP	Mamu-E0201_R	Mamu-E0201_F	HLA-E_mamu0216W->G_EGFP	HLA-E_mamu0216W->G_EGFP
HLA-E_mamu0201G->W_EGFP	Mamu-E0201_R	Mamu-E0201_F	HLA-E_mamu0216_EGFP	HLA-E_mamu0216_EGFP
HLA-E_mamu0204_SN_EGFP	SN_R	SN_F	HLA-E_mamu0204_EGFP	HLA-E_mamu0204_EGFP
HLA-E_mamu0221_EGFP	Mamu-E0221_R	Mamu-E0221_F	HLA-E_mamu0204_SN_EGFP	HLA-E_mamu0201_EGFP
HLA-E_mamu0221W->G_EGFP	Mamu-E0221_R2	Mamu-E0221_F2	HLA-E_mamu0221_EGFP	HLA-E_mamu0221_EGFP
pLenti HLA-E_APEX2_flag	E_BamHI_F /E_APEX2_R	E_APEX2_F/ APEX2_flag_SalI_R	HLA-E_EGFP	APEX2-NLBP3-flag
pLenti HLA-EA3_APEX2_flag	E_BamHI_F /A3_APEX2_R	A3_APEX2_F/ APEX2_flag_SalI_R	HLA-EA3_EGFP	APEX2-NLBP3-flag
pLenti HLA-A3_APEX2_flag	A3_BamHI_F /A3_APEX2_R	A3_APEX2_F/ APEX2_flag_SalI_R	HLA-A3_EGFP	APEX2-NLBP3-flag
pLenti HLA-A3E_APEX2_flag	A3_BamHI_F /E_APEX2_R	E_APEX2_F/ APEX2_flag_SalI_R	HLA-E_EGFP	APEX2-NLBP3-flag

Table S4 Antibody list

antibody	target	concentration (ng/μl)/dilution	assay	source
APC-conjugated 3D12	HLA-E constructs	2.5	FC	BioLegend, 342606
APC-conjugated GAP.A3	HLA-A3 constructs	2.5	FC	Life Technologies, 17-5754-42
Unconjugated 3D12	HLA-E constructs	2.5	FC	BioLegend, 342602
APC-conjugated anti-mouse	Mouse IgG	0.2	FC	Invitrogen, 17-4010-82
Goat anti-mouse Alexa Fluor 647	Mouse IgG	1	IF	Invitrogen, A32728
OTI1F10	AP2M1	1	WB	Life Technologies, MA5-25570
anti-CLTC	CLTC	0.5	WB	Proteintech, 26523-1-AP
EPR3307(2)	VCP	1:2000	WB	Abcam, ab109240
GA1R	GAPDH	0.2	WB	Life Technologies, MA5-15738
anti-GAPDH	GAPDH	0.2	WB	Sigma-Aldrich, G9545
IRDye 680RD goat anti-mouse	Mouse IgG	1:10000	WB	LI-COR, 926-68070
IRDye 800CW Streptavidin	biotin	1:10000	WB	LI-COR, 926-32230
IRDye 800CW donkey anti-mouse	mouse IgG	0.05	WB	LiCor, P/N: 926-32212,
anti-RAB7	RAB7	0.05	IF	Abcam, ab137029
anti-RAB11	RAB11	1	IF	Life Technologies, 71-5300
goat anti-rabbit Alexa Fluor 568	rabbit IgG	2	IF	Abcam, ab175471
anti-FLAG tag	FLAG tag	1	IF	Sigma-Aldrich, F1804
		0.5	WB	
Alexa Fluor 488 conjugated streptavidin	biotin	0.5	IF	Invitrogen, S32354
AP.6	AP1A1	5	ELISA	Produced in lab
HRP-linked anti-mouse antibody	mouse IgG	1:2000	ELISA	Cell Signaling, 7076

*FC, flow cytometry; WB, western blotting; IF, immunofluorescence; ELISA, enzyme-linked immunosorbent assay.
