# Supplementary figures and images for: A cytoplasmic motif in HLA-E that drives clathrin-mediated endocytosis and VCP-associated postendocytic trafficking
Source: Proc Natl Acad Sci U S A. 2025 Oct 24;122(43):e2514956122. doi: 10.1073/pnas.2514956122 (PMC12582296; doi:10.1073/pnas.2514956122)

datafile.S1. original blots for Figure 6

cropping zone

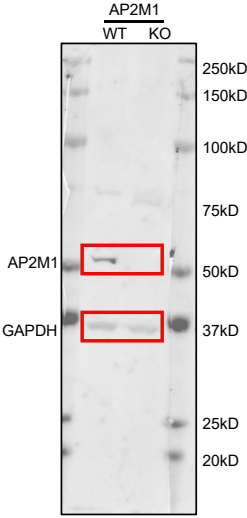

(Figure 6a)

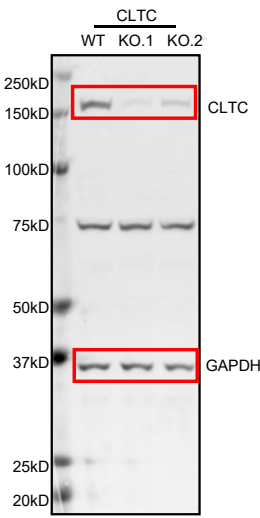

(Figure 6c)

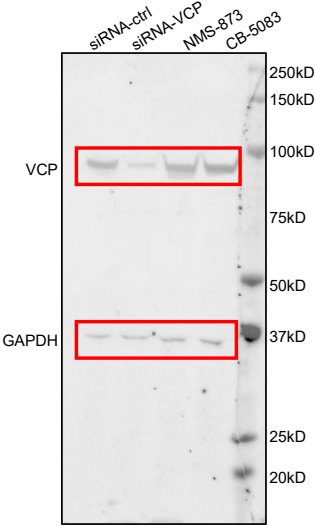

(Figure 6h)

Supplement: Supplementary file 2 — Dataset S01 (PDF) [file pnas.2514956122.sd01.pdf]
